# Evolution of Multivalent Aptamer Corona for High‐Throughput Multiplexed Detection of Multiple Cancers

**DOI:** 10.1002/advs.202514976

**Published:** 2025-11-25

**Authors:** Mengjie Wang, Baichuan Jin, Xin Dai, Haozhe Huang, Qiqi Liu, Jianqing Zhu, Haixing Ju, Qixun Chen, Yongmei Song, Weihong Tan, Yuan Liu

**Affiliations:** ^1^ Institute of Department Molecular Science and Biomedicine Laboratory (MBL) State Key Laboratory of Chemo/Bio‐Sensing and Chemometrics College of Chemistry and Chemical Engineering Aptamer Engineering Center of Hunan Province Hunan University Changsha Hunan 410082 China; ^2^ Institute of Zhejiang Cancer Hospital Hangzhou Institute of Medicine (HIM) Chinese Academy of Sciences Hangzhou Zhejiang 310022 China; ^3^ Institute of Cancer Centre and Institute of Translational Medicine Faculty of Health Sciences University of Macau Macau SAR 999078 China; ^4^ State Key Laboratory of Molecular Oncology National Cancer Center/National Clinical Research Center for Cancer/Cancer Hospital Chinese Academy of Medical Sciences and Peking Union Medical College Beijing 100021 China

**Keywords:** aptomics, multivalent aptamer corona, multiplexed cancer diagnosis, proteomics, ProteoFish‐SELEX

## Abstract

Clinical in vitro diagnosis is essential in early diagnosis and prognostic assessment, yet antibody‐based assays face limitations in multiplexity and scalability. To address the growing demand for modern molecular diagnostics, a multivalent aptamer corona platform for high‐throughput is introduced and multiplexed multi‐cancer diagnosis. With clinical serum samples, ovarian cancer‐, lung cancer‐, and colorectal cancer‐based aptamer coronas are obtained through several rounds of alternating positive and negative systematic evolution of ligands by exponential enrichment (SELEX). Leveraging a de novo ProteoFish‐SELEX strategy, which integrates nanoparticle‐protein corona technology, a multivalent aptamer corona is evolved. Proteomic profiling of protein coronas revealed cancer‐associated protein signatures, while aptomic profiling of aptamer coronas identified high‐affinity cancer‐specific aptamers. With these aptamers, high diagnostic accuracy is demonstrated in multiplexed multi‐cancer detection using clinical cohorts. Multivalent aptamer corona's outstanding diagnostic performance, multiplexed detection capability, and sequencing‐enabled high‐throughput potential position it as a promising versatile tool for novel biomarker discovery and a transformative advancement in precision oncology.

## Graphical abstract

A multivalent aptamer corona is evolved via established ProteoFish‐SELEX (systematic evolution of ligands by exponential enrichment) for high‐throughput multiplexed detection of multiple cancers. Clinical validation using multivalent aptamer corona demonstrated high accuracy and specificity in multiplexed detection of ovarian, lung, and colorectal cancers using serum samples from different cohorts.

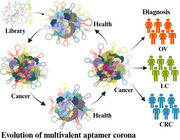

## 1 Introduction

Blood‐based in vitro diagnosis (IVD) is a cornerstone for modern clinical practice owing to its non‐invasiveness, simplicity, and scalability in large‐scale population screening.^[^ ^1^, ^2^, ^3^ ^]^ By detecting disease‐specific biomarkers in blood, IVD enables early diagnosis, prognosis monitoring, and therapeutic response assessment, making it essential for precision medicine.^[^ ^4^, ^5^, ^6^, ^7^ ^]^ Currently, blood biomarker detection predominantly relies on antibody‐based immunoassays. Although they offer high specificity and sensitivity, these platforms exhibit critical limitations.^[^ ^8^ ^]^ For instance, multiplexed biomarker analysis requires parallel workflows, which have higher costs and prolonged turnaround times since antibodies typically support single‐plex detection per analytical run.^[^ ^9^ ^]^ In addition, multi‐cancer screening in clinical application is significantly hindered by the limited FDA‐approved blood‐based cancer biomarkers and the persistent issues in assay reproducibility from antibody inter‐batch variability.^[^ ^10^, ^11^, ^12^ ^]^ Furthermore, it is extremely challenging to detect low‐abundant biomarkers due to the matrix interference proteins in complex blood milieux.^[^ ^13^ ^]^

To address these limitations and reduce the antibody dependence, nucleic acid aptamers and advanced nano‐biosensors have emerged as promising platforms for specific and sensitive biomarker detection.^[^ ^14^, ^15^ ^]^ Aptamers, selected through systematic evolution of ligands by exponential enrichment (SELEX),^[^ ^16^, ^17^ ^]^ exhibit inherent advantages over antibodies, including minimal batch‐to‐batch variability, compact size and structural adaptability, ease of site‐specific chemical modification, and cost‐effective production.^[^ ^18^, ^19^, ^20^, ^21^ ^]^ Despite these merits, aptamer‐based multiplexed detection of blood biomarkers is unwonted owing to its deterioration in blood.^[^ ^22^, ^23^, ^24^ ^]^ Unlike antibodies, rapid enzymatic degradation and hydrolysis of unmodified aptamer in complex biological matrices have hindered their robust application in clinical diagnostic settings.^[^ ^25^, ^26^ ^]^

The formation of nanoparticle‐protein corona (NPC) upon incubating nanoparticles with biological fluid represents a significant advancement for biomarker discovery and detection.^[^ ^27^, ^28^, ^29^ ^]^ Especially, NPC enriching low‐abundance proteins, including cancer‐specific protein biomarkers, has been demonstrated to be able to differentiate lung cancer from healthy controls.^[^ ^30^, ^31^, ^32^ ^]^ Aptamers that can selectively recognize the cancer‐associated protein targets within NPC provide a compelling strategy for blood‐based in vitro diagnosis.^[^ ^33^, ^34^ ^]^ However, although numerous aptamers have been developed against cell‐surface targets via cell‐SELEX, few sequence‐specific aptamers targeting soluble blood proteins have been reported for liquid biopsy applications,^[^ ^35^ ^]^ highlighting a critical gap in diagnostic aptamer development. Inspired by this capability, we conceived a SELEX platform that directly exploits the proteomic complexity of clinical serum as preserved within the NPC to overcome the constraints of cell‐based methods, thereby enabling the evolution of clinically relevant aptamers for liquid biopsy. In this framework, proteomics entails the large‐scale analysis of proteins adsorbed onto nanoparticles, while aptomics refers to the high‐throughput profiling of aptamer sequences selected against the corona. This approach effectively addresses the critical need for multiplexed, high‐affinity aptamer panels, positioning it as a highly promising strategy for advancing multi‐cancer serological diagnosis.

**Figure 1:**
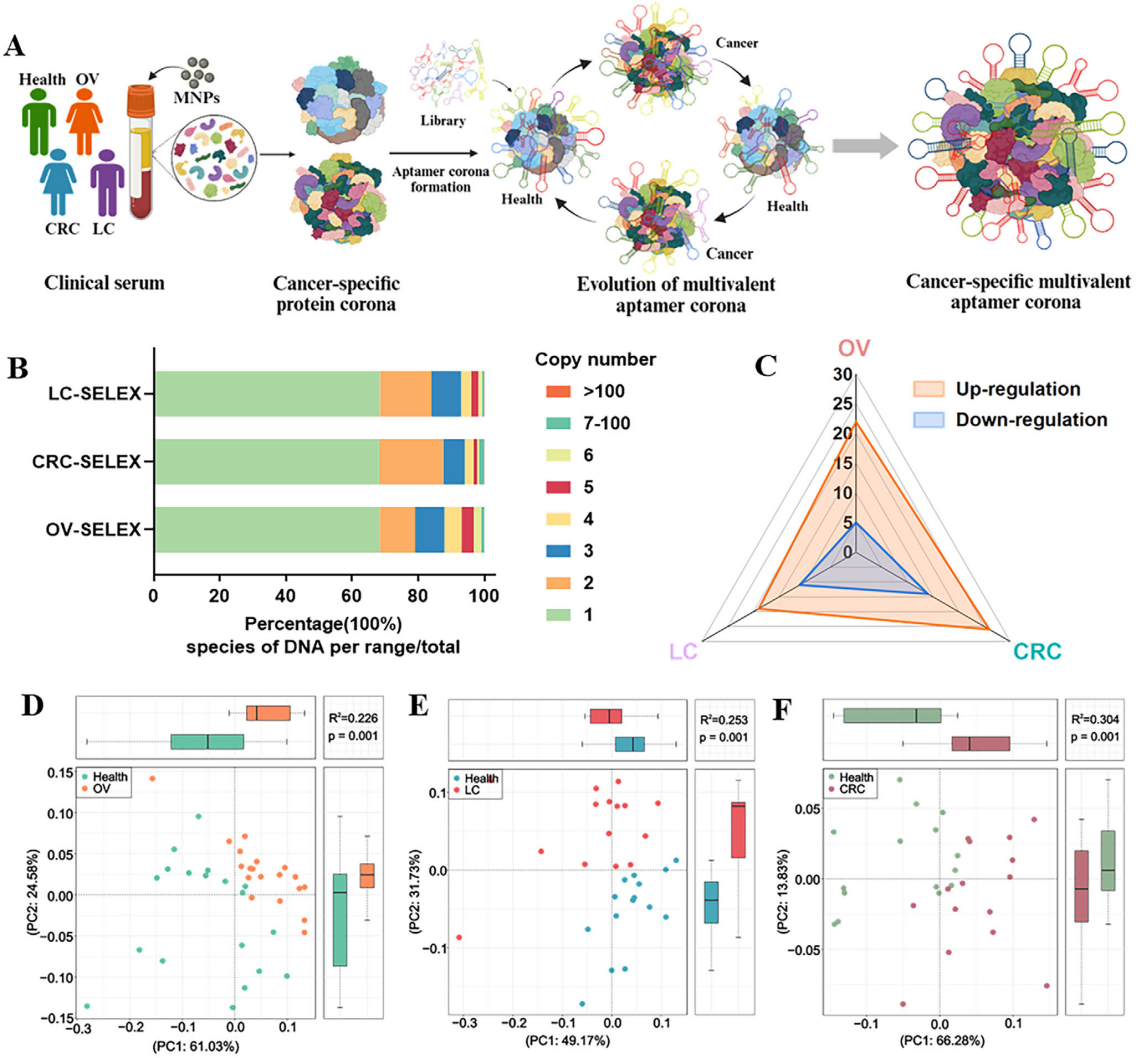
Evolution of multivalent aptamer corona via ProteoFish‐SELEX. A) Workflow of the ProteoFish‐SELEX process for evolving cancer‐specific multivalent aptamer coronas. Key steps include: (1) formation of NPCs; (2) negative selection step against healthy NPCs; (3) iterative positive selection against cancer NPCs; and (4) NGS of the enriched aptamer pools to reveal candidate sequences. Created in https://BioRender.com. B) Copy number distribution of aptamer corona from the last round enriched pool in different cancer types. Distinct colors represent different copy numbers. C) Radar chart displaying the count of DEAs identified for each cancer type versus healthy controls. Upregulated and downregulated DEAs are shown in orange and blue, respectively, revealing cancer‐specific signatures for OV (27 sequences), LC (30 sequences), and CRC (40 sequences). D–F) PCA plot analysis of clinical serum cohort validation using DEAs of aptamer corona from (D) OV (*n* = 48), (E) LC (*n* = 30), and (F) CRC (*n* = 30), respectively.

In this study, we evolved a multivalent aptamer corona via ProteoFish‐SELEX platform for multiplexed multi‐cancer diagnosis (**Figure** **1**A). Clinical cancer serum (ovarian cancer, Lung cancer, and colorectal cancer) was collected to incubate with nanoparticles for the formation of cancer‐specific NPC. Aptamer library, a pool containing 10^18^ different aptamer sequences, was then incubated with NPC to form an aptamer corona. Serum from healthy controls generated NPC was used for negative SELEX. With alternative positive‐ and counter‐SELEX, cancer‐specific aptamers and protein targets were identified via sequencing and mass spectrometry as the evolution of aptamers by exponential enrichment. With a selected cancer‐specific aptamer panel, a multivalent aptamer corona was established for blood‐based multi‐cancer diagnosis.

## 2 Results and Discussion

### 2.1 Evolution of Multivalent Aptamer Corona via ProteoFish‐SELEX Platform

To establish the ProteoFish‐SELEX platform, we employed Fe_3_O_4_@SiO_2_ magnetic nanoparticles (MNPs) capable of streamlining NPC isolation via magnetic separation.^[^ ^36^, ^37^ ^]^ Post‐ProteoFish processing induced a modest size increase in MNPs, which is consistent with protein corona formation (Figure S1, Supporting Information). Previous research has established that NPCs formed in clinical serum encapsulate patient‐specific molecular signatures, thereby preserving disease‐relevant pathological information.^[^ ^38^, ^39^, ^40^ ^]^ To mitigate inter‐individual variability, we randomly selected 6 serum samples from 6 different subjects and pooled them into a single aliquot. We scaled this strategy into six parallel aliquots, each containing pooled samples from six unique cancer patients (total *n* = 36) for one single positive‐SELEX pipeline (Figure S2, Supporting Information). For complementary counter‐SELEX, an equivalent cohort of healthy donors (*n* = 36) underwent identical pooling to eliminate nonspecific binders. This pooled serum design ensured the generation of broadly generalizable aptamers robust against biological heterogeneity. The ProteoFish‐SELEX workflow commenced with a negative selection round using serum samples from healthy subjects. NPCs were formed by incubating MNPs with serum, followed by washing to remove unbound proteins. A single‐stranded DNA library with 66 nucleotides (18‐nt primers flanking a 30‐nt randomized region) was then introduced into NPCs, enabling the formation of aptamer corona through sequence‐target binding. The unbound sequences from six parallel negative selection workflows were pooled and advanced to the positive‐SELEX phase. For positive selection, NPCs were reconstituted using serum samples from cancer patients, preserving disease‐specific proteomic complexity. Aptamer candidates surviving counter‐SELEX were incubated with cancer serum‐derived NPCs to form target‐specific multivalent aptamer corona. Nonbinding sequences were removed through stringent washing, while high‐affinity aptamers that bound to target proteins were recovered via elution. Six or seven cycles of negative‐ and positive‐SELEX were performed, after which the final enriched aptamer pool from the last positive selection round underwent next‐generation sequencing to identify candidate sequences.

Upon establishing the ProteoFish‐SELEX platform, we assessed its capacity in identifying cancer‐specific biomarkers and their cognate aptamers with clinical serum cohorts encompassing ovarian cancer (OV), lung cancer (LC), and colorectal cancer (CRC), alongside healthy donor controls using the same DNA library (Tables S3 and S4, Supporting Information). Iterative SELEX rounds revealed distinct amplification curve dynamics, showing that fluorescence intensity stabilized and plateaued after 3 to 5 cycles, indicating a marked reduction in DNA library diversity (Figure S3A–C, Supporting Information). Parallel analysis of melting curves showed progressive migration of dominant peaks across successive rounds, demonstrating the iterative refinement of high‐affinity aptamers (Figure S3D–F, Supporting Information). The metrics of amplification, plateau phase, and peak migration collectively validated aptamer convergence during the evolution of aptamer corona,^[^ ^41^, ^42^ ^]^ confirming the platform's ability to isolate sequence pools with increased specificity for cancer‐associated epitopes. Quantitative analysis of sequencing data from SELEX‐enriched pools of OV, LC, and CRC, respectively revealed a strikingly low proportion (≈0.1%) of highly enriched aptamers (copy number >100) in the final rounds: 0.09% (OV), 0.11% (CRC), and 0.10% (LC) (Figure 1B). These results suggest that ProteoFish‐SELEX can effectively distinguish specific binding sequences from nonspecific adsorption sequences, thereby facilitating the identification of corona aptamer candidates.

**Figure 2:**
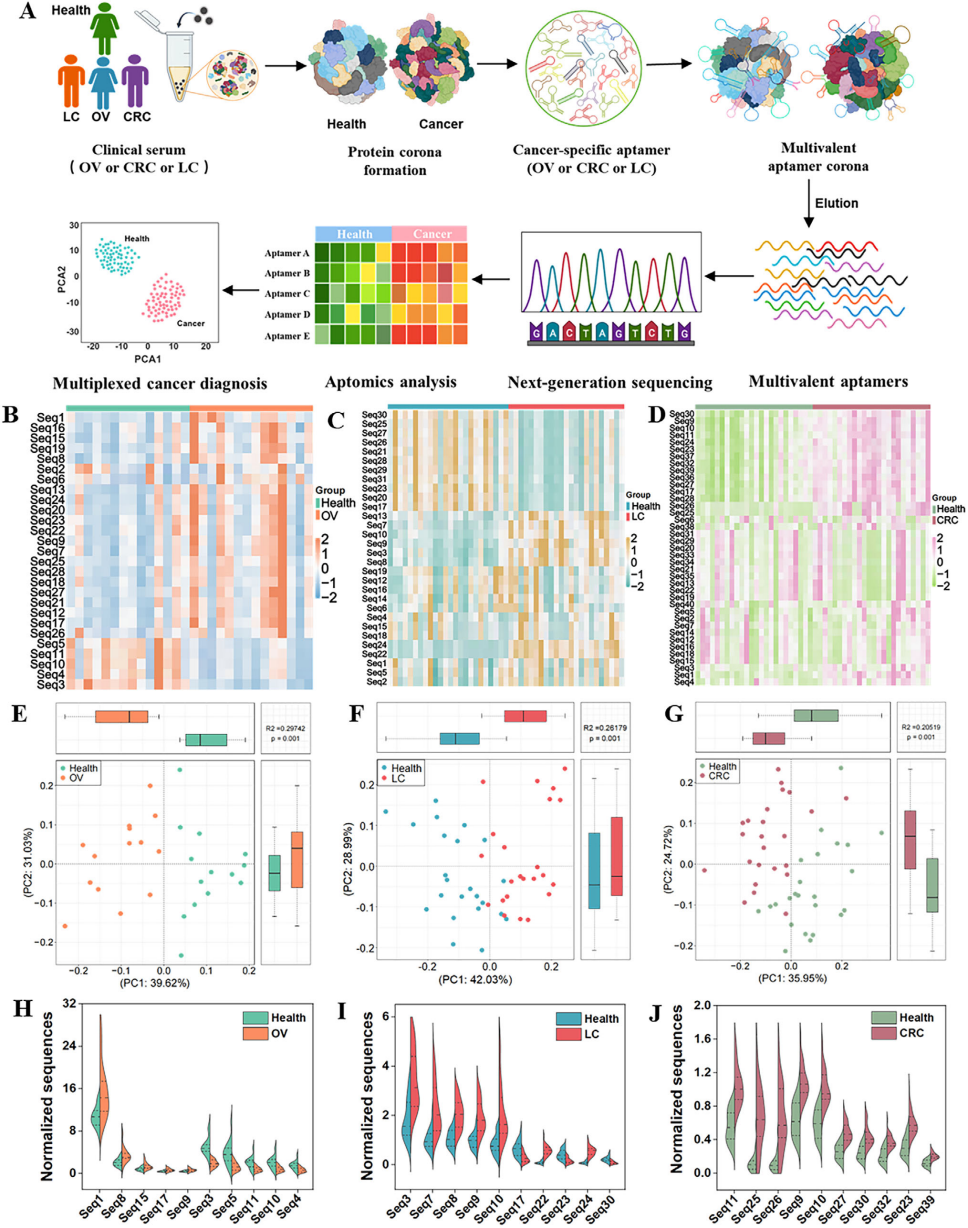
Discrimination validation of distinct cancers using synthetic differentially expressed aptamers (DEAs). (A) Schematic workflow of the diagnostic validation pipeline. The binding specificity of the individually synthesized DEA panels (OV: 27 sequences; LC: 30 sequences; CRC: 40 sequences) was validated by incubating each with type‐matched NPCs from an independent cohort. Bound aptamers were eluted and quantified via high‐throughput sequencing. B–D) Hierarchical clustering of synthetic DEA binding profiles reveals a clear separation between cancer patients and healthy controls in the independent validation cohorts for (B) OV (*n* = 28), (C) LC (*n* = 30), and (D) CRC (*n* = 30). Rows represent individual aptamers, columns represent serum samples. E–G) PCA based on synthetic DEA binding profiles demonstrates clear separation between cancer samples (red) and healthy controls (blue) for (E) OV, (F) LC, and (G) CRC, confirming the robust diagnostic power of the selected aptamers. H–J) Relative expression levels of the top‐10 DEA aptamers (normalization against the internal standard reference sequence was applied) in OV vs healthy control (H), LC vs healthy control (I), and CRC vs healthy control (J).

The three final round evolved aptamer coronas were amplified via PCR and assessed for their ability to discriminate cancer cohorts (OV: *n* = 36; LC: *n* = 30; CRC: *n* = 30) from healthy controls in a preliminary diagnostic evaluation. The three amplified aptamer coronas were first incubated with healthy and cancer‐specific NPCs to form aptamer coronas and then eluted for aptamer sequencing (**Figure** **2**A). Comparative analysis of eluted aptamers revealed no significant differences in quantity between cancer cohorts and healthy controls (Figure S4, Supporting Information). However, principal component analysis (PCA) of bound aptamer profiles achieved robust separation between cancer groups and healthy controls (Figure S5B,D,F, Supporting Information). While, hierarchical clustering heatmap analysis showed no distinct clusters as a result of background signal interference (Figure S5A,C,E, Supporting Information).

Bioinformatics profiling of differentially expressed aptamers (DEAs) isolated from the final round of SELEX‐enriched pools identified cancer‐specific signatures, including 27 sequences for OV, 30 sequences for LC, and 40 sequences for CRC (Figure S6 and Tables S5–S7, Supporting Information). Notably, upregulated aptamers predominated over downregulated counterparts in each panel (Figure 1C). DEA‐based stratification achieved clear discrimination between cancer cohorts and healthy controls (Figure 1D–F), and heatmap patterns corroborated PCA‐derived separations (Figure S7, Supporting Information). Phylogenetic reconstruction using MEGA software delineated distinct aptamer families unique to each cancer type (Figure S8, Supporting Information), while pairwise Pearson correlation analysis confirmed significant dissimilarities in sequence profiles across OV, LC, and CRC (Figure S9, Supporting Information). Collectively, these findings underscore the diagnostic superiority of DEA panels over unselected enriched pools for cancer discrimination.

### 2.2 Multivalent Aptamer Corona Utilizing DEAs for Cancer Diagnosis

To further validate DEA specificity and robustness, freshly synthesized aptamers of three DEA panels (OV: 27 sequences; LC: 30 sequences; CRC: 40 sequences) were independently tested against cancer‐matched NPCs using three new cohorts (OV: *n* = 28, LC: *n* = 48, CRC: *n* = 48). For OV, an equimolar mixture of synthetic OV‐DEA was incubated with OV‐derived NPCs to form a multivalent aptamer corona, followed by sequencing the eluted aptamers (Figure 2A). Parallel experiments were conducted for LC and CRC with their corresponding DEA panels and derived NPCs, respectively. Freshly synthesized DEAs achieved better diagnostic discrimination than that of the whole enriched pools, owing to the excluded background sequences. We observed a clear distinction between the healthy and OV groups from PCA and heatmap (Figure 2B,E). Similarly, two clear clusters were observed from PCA and heatmap in LC and CRC validation (Figure 2C,D,F,G).

Further refinement of three distinct top 10 aptamer subpanels from OV‐DEA, LC‐DEA, and CRC‐DEA, respectively, which were ranked by *p*‐value within each cancer group, surpassed both full DEA sets and enriched pools (Figure S10A–C, Supporting Information), as validated by enhanced PCA separation (Figure S10D–F, Supporting Information) and heatmap resolution (Figure S10G–I, Supporting Information). In addition, the individual aptamer level of the top 10 selected aptamers was shown in Figure 2H‐J. Overall, the validation using independent clinical cohorts demonstrated that the evolved multivalent aptamer corona with specific DEAs has great potential in cancer diagnosis.

Furthermore, we evaluated the time‐dependent degradation of the aptamer adsorbed onto the NPC surface and compared it to the aptamer incubated in serum. As shown in Figure S11 (Supporting Information), the integrity of the serum‐incubated aptamer (Seq2 in OV) decreased over time, whereas the aptamer bound to the NPC surface remained intact. These results indicate that the aptamer corona effectively shields the aptamers from enzymatic degradation.

### 2.3 Proteomic Profiling of Nanoparticle‐Protein Corona to Identify Cancer‐Associated Proteins

**Figure 3:**
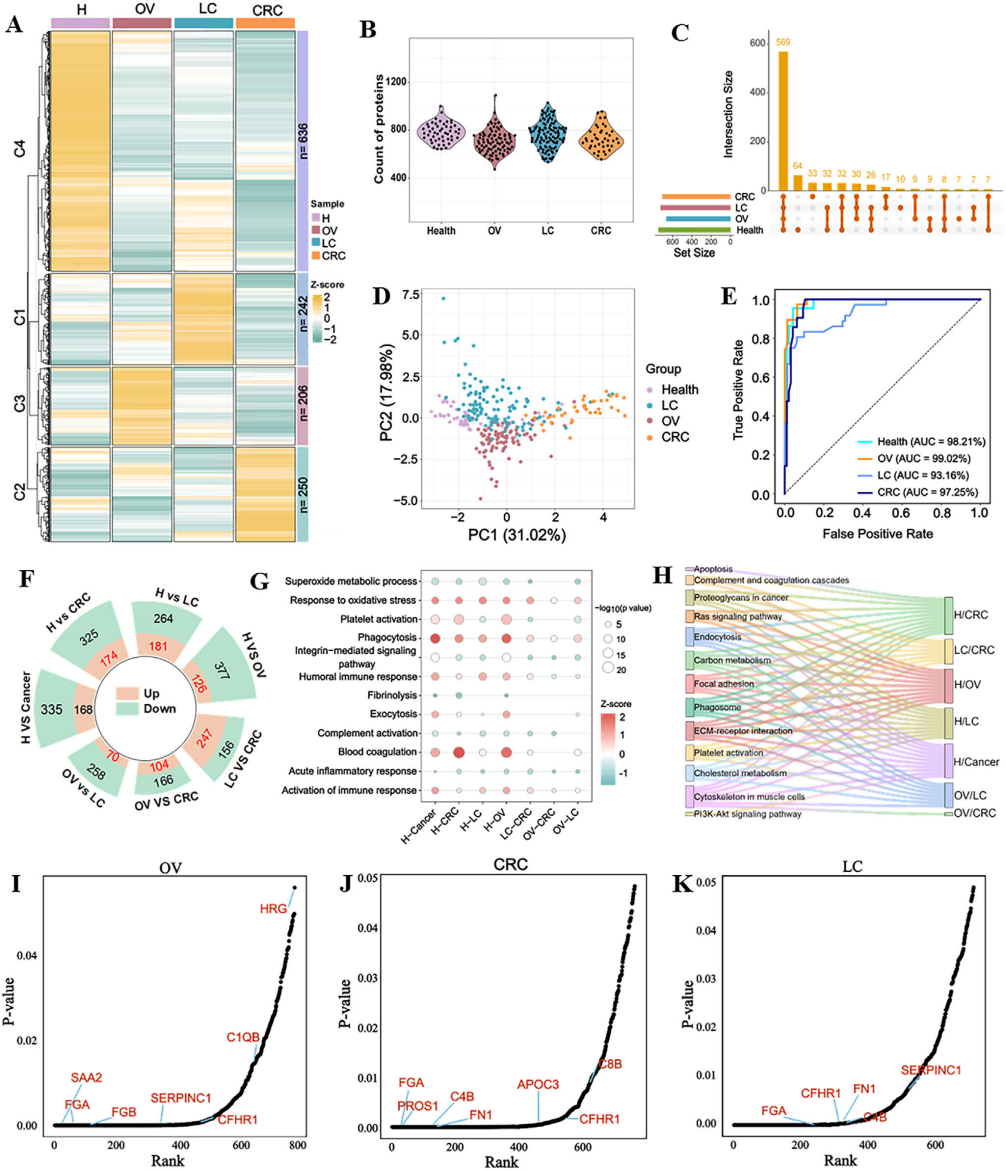
Proteomic profiling of different cancer cohorts. A) Heatmap of 1338 variably expressed proteins by K‐means clustering via ProteoFish from three different cancer types. Proteins were grouped into four clusters on the basis of expression similarity. The number of expressed proteins in each cluster is indicated. B) Number of proteins identified in each cancer type via ProteoFish. C) Upset plot showing unique and common proteins in different cancers. The top bar graph shows the number of proteins identified for each cancer type. D) PCA plot using the selected top‐10 key proteins, indicating an obvious discrimination of OV, LC, CRC, and healthy control. E) ROC‐AUC plots showing discrimination performance for each cancer group with the selected top‐10 key proteins by the random forest model. F) Radial stacked plots listing the differentially expressed protein numbers of distinct pairwise comparisons (fold change > 1.5 and adjusted *p*‐value < 0.05). Pink color denotes upregulated proteins, and green color denotes downregulated proteins. G) Dot plot representation of dysregulated pathways enriched in GO across multiple cancer comparisons. The size of the circle denotes the value of ‐log10(P), and the color represents the enrichment significance. H) A Sankey diagram shows the pathways of differentially expressed proteins in KEGG by comparison of different cancers. I–K) Rank of differentially expressed proteins for OV‐derived NPC (I), LC‐derived NPC (J), and CRC‐derived NPC (K), as profiled by LC‐MS/MS. Annotated proteins are those identified with specific aptamers.

Cancer‐associated proteins within the protein corona lay the foundation for the aptamer corona to differentiate the cancer serum from healthy controls. To delineate the proteomic landscape underlying the aptamer corona interactions, we performed LC‐MS/MS profiling of NPCs derived from clinical cohorts (OV: *n* = 87; LC: *n* = 114; CRC: *n* = 41; Healthy control: *n* = 50; Figure S12 and Table S1, Supporting Information). In total, 1338 proteins were identified across 292 subjects, with mean protein groups per cohort ranging from 706 to 781 (**Figure** **3**B) and substantial overlap among cancer types (Figure 3C).

Proteomic analysis demonstrated that the ProteoFish platform significantly remodeled the serum protein composition. Notably, human serum albumin (ALB), the most abundant serum protein, showed a marked decrease in its relative abundance, dropping from 40.61% to 0.56%, accompanied by a drop in its abundance rank from 1st to 37th (Table S8, Supporting Information). In addition, the cumulative proportion of the top 10 proteins decreased from 72.89% in serum to 52.15% in the NPC (Figure S13, Supporting Information). This effective depletion of high‐abundance proteins and the consequent enrichment of low‐abundance proteins enabled deep proteomic profiling of serum by LC‐MS/MS. K‐means clustering partitioned the proteome into four distinct clusters, revealing cancer‐associated enrichment of specific protein subsets (Figure 3A; Figure S14A–D, Supporting Information). A random forest classifier identified a 10‐protein panel that robustly discriminated cancer types (AUC >0.90; Figure 3D,E; Figure S14E,F, Supporting Information) with differential expression confirmed across 4 cohorts (Figure S15, Supporting Information). These findings validate that the NPC serves as a proteomic reservoir enriched with many cancer‐associated protein signatures, thus enabling aptamer corona recognition and diagnostic stratification.

Bioinformatics prioritization of differentially expressed proteins (DEPs) across cohorts revealed cancer‐specific proteomic signatures through pairwise group comparisons (Figure 3F; Figure S16, Supporting Information). DEPs in OV, LC, and CRC were ranked by statistical significance (*p*‐value; Figure 3I‐K). Serum amyloid A2 (SAA2), a pan‐cancer upregulated protein linked to tumor pathogenesis,^[^ ^43^, ^44^ ^]^ emerged as a dominant biomarker across healthy/cancer (H/Cancer), H/CRC, H/LC, and H/OV contrasts. ProteoFish‐SELEX further validated this linkage by isolating a high‐affinity SAA2 aptamer, demonstrating that DEPs serve as direct molecular targets for aptamer binding and diagnostic stratification (Figure S19E, Supporting Information). This synergy between proteomic biomarker discovery (DEPs) and aptamer screening establishes a pipeline to translate protein biomarker detection into aptamer corona‐based diagnostic platforms.

Pathway enrichment analysis (Gene Ontology and KEGG) was employed to delineate the biological significance of DEPs in different cancer cohorts. Dysregulated pathways linked to coagulation, phagocytosis, platelet activation, oxidative stress response, and immune activation were recurrently enriched in cancer groups (Figure 3G), consistent with their roles in tumor‐associated coagulation dysfunction and metastatic progression.^[^ ^45^, ^46^ ^]^ Conversely, metabolic regulation, complement activation, and acute inflammatory response pathways were suppressed (Figure 3G), reflecting immunosuppressive mechanisms characteristic of cancer biology.^[^ ^47^, ^48^ ^]^ KEGG analysis further highlighted perturbations in platelet activation, ECM‐receptor interactions, and Ras signaling, underscoring NPC as a reflection of both oncogenic signaling and microenvironmental adaptation (Figure 3H). Proteomic profiling of NPCs indicated that cancer‐associated proteins are selectively adsorbed onto nanoparticles via ProteoFish, enabling direct biomarker discovery on NPC surfaces. This integration of NPC‐based proteomics and pathway analysis establishes a platform for identifying aptamer‐bound cancer‐associated protein targets, highlighting the potential of the aptamer corona strategy for non‐invasive cancer diagnosis.

### 2.4 Aptomic Profiling of Aptamer Corona Across Multiple Cancers to Identify Cancer‐Specific Aptamers

**Figure 4:**
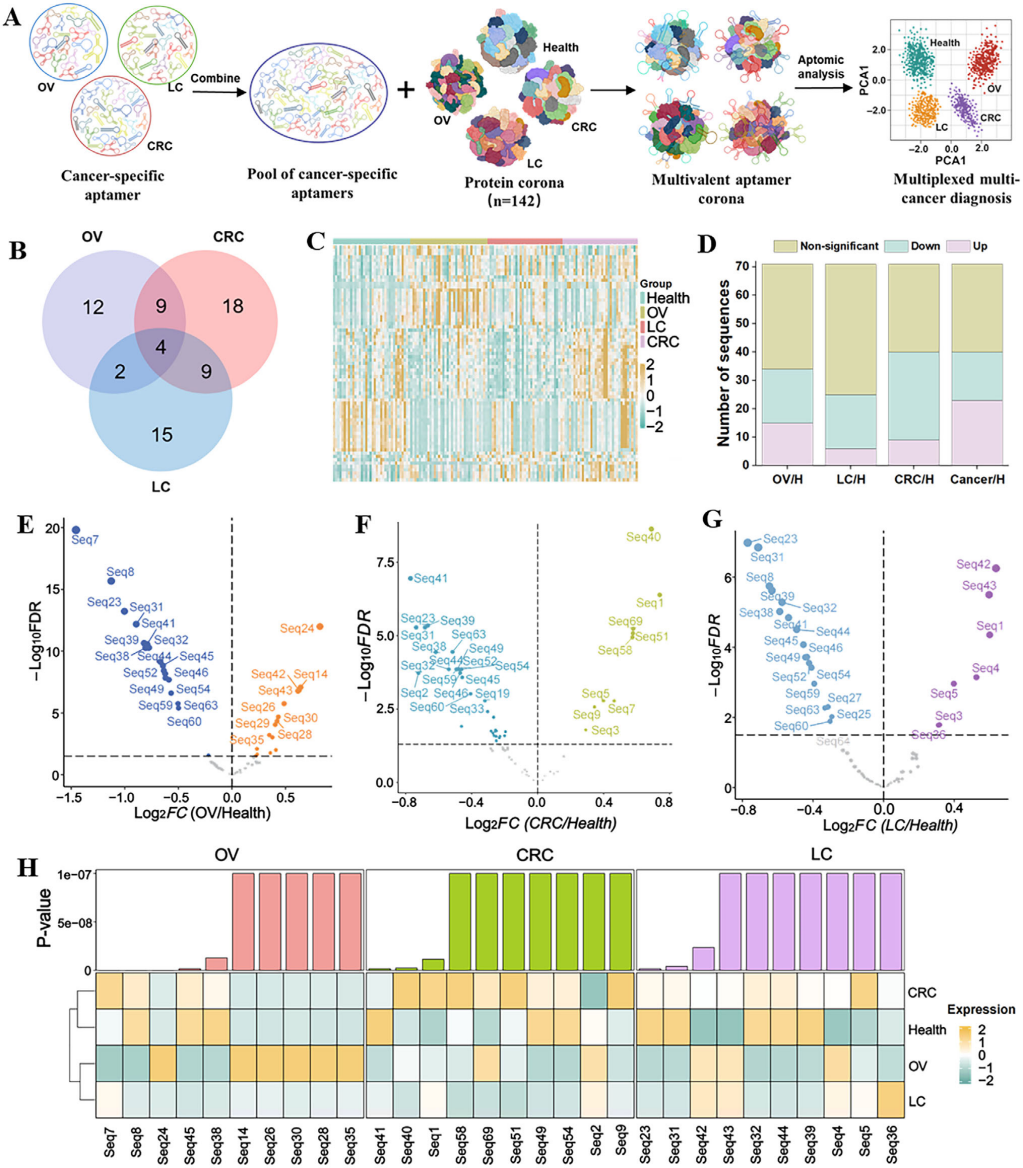
Cancer‐specific aptamer identification for multivalent aptamer corona. A) Workflow of cancer‐specific aptamer identification using combined and refined DEAs from different cancers. DEAs from individual OV (27 sequences), LC (30 sequences), and CRC (40 sequences) screenings were merged into a unique 69‐aptamer panel. This panel was incubated with NPCs from an independent cohort (*n* = 142), and bound aptamers were eluted and quantified via NGS using an internal standard for normalization. B) Venn diagram showing the number of common and unique DEA aptamers of different cancers. C) Heatmap showing aptamer expression level from 142 subjects, including OV, CRC, LC, and healthy controls. D) Summary of the number of upregulated, downregulated, and nonsignificant aptamers for different comparison groups: OV/H, LC/H, CRC/H, and overall cancer/H. E–G) Volcano plots showing the differentially expressed aptamers for OV (E), CRC (F), and LC (G). Downregulated and upregulated aptamers were annotated in plots. H) Aptamer sequencing quantification‐based expression profile of the top 10 DEA aptamer panels with the most significant *p*‐values in different cancers.

To evaluate the multi‐cancer diagnostic capability of aptamer corona, we curated a composite aptamer panel comprising all DEAs selected from the final round positive‐SELEX of OV (27 sequences), LC (30 sequences), and CRC (40 sequences). Venn diagram revealed 4 shared aptamers across all three cancers, alongside 12 OV‐specific, 15 LC‐specific, and 18 CRC‐specific sequences (**Figure** **4**B). After deduplication, a refined panel of 69 unique aptamers was synthesized and pooled to a single aliquot at equimolar ratios for downstream discrimination validation (Table S9, Supporting Information). This panel was tested against an expanded independent clinical cohort (*n* = 142), including 36 OV, 35 LC, 35 CRC, and 36 healthy control subjects with demographic and staging information detailed in Figure S15 and Table S2 (Supporting Information). Serum‐derived NPCs were generated via ProteoFish by incubating MNPs with cohort serum. The pooled 69‐aptamer panel was exposed to NPCs to form a multivalent aptamer corona, followed by binding aptamer elution (Figure 4A). To ensure sequencing fidelity, a standardized reference sequence was spiked into each eluted sample (*n* = 142) prior to next‐generation sequencing. Normalization against this internal standard enabled robust cross‐sample quantification (Figure 4C).

With normalized aptamer abundance profiles established, we systematically evaluated differential binding dynamics of aptamers to corona proteins (Figure 4D). Venn analysis identified cancer‐unique DEAs (OV: 2; LC: 3; CRC: 12), alongside 14 shared candidates, with different expression profiles detailed in Figure S16 (Supporting Information). Volcano plots further delineated reclassified DEAs across OV, CRC, and LC cohorts (Figure 4E–G), while a hierarchical clustering heatmap of the top 10 DEAs revealed cancer‐specific aptamer abundance patterns (Figure 4H).

### 2.5 Multivalent Aptamer Corona for High‐Throughput Multiplexed Multi‐Cancer Diagnosis

**Figure 5:**
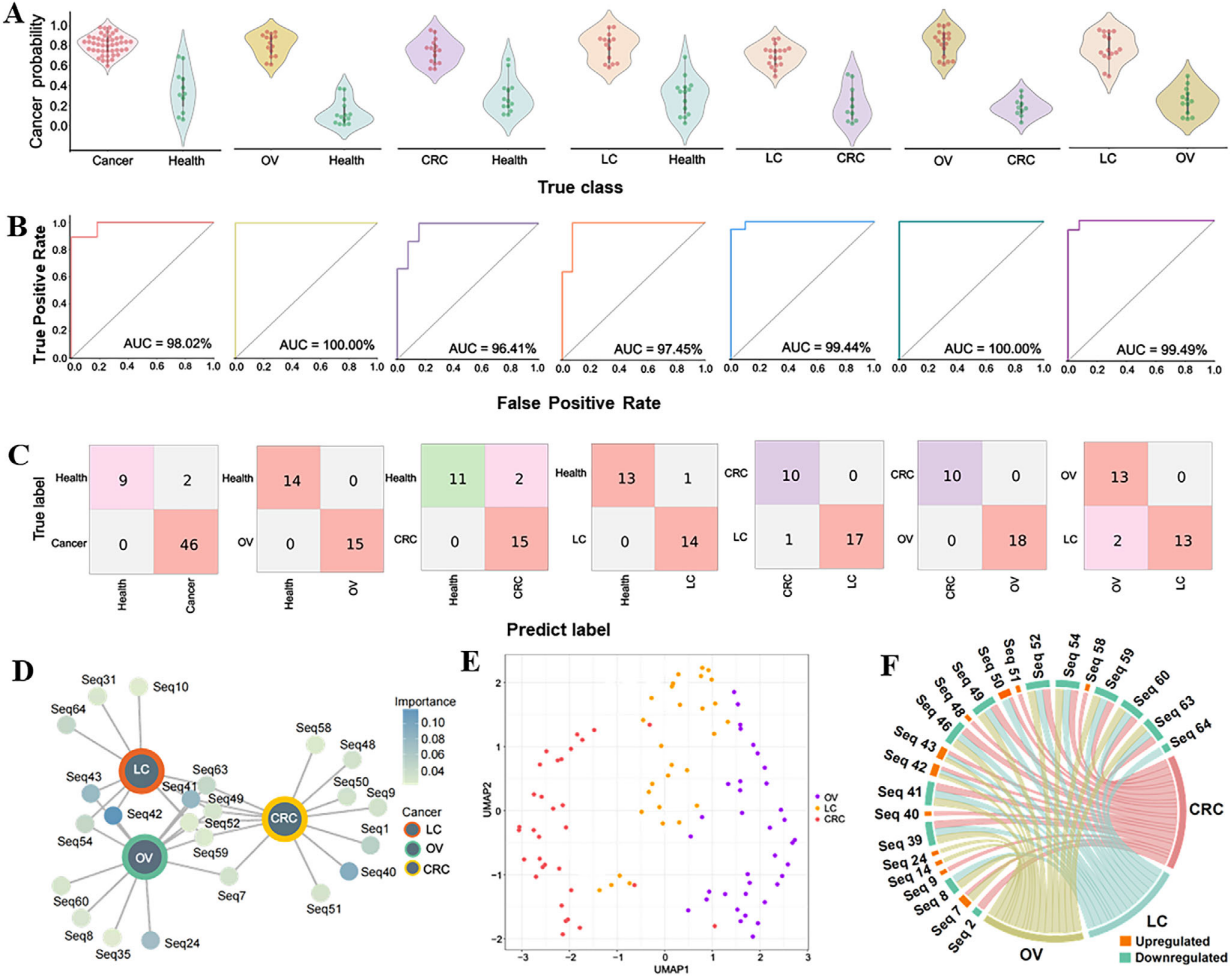
Discrimination performance using a multivalent aptamer corona with cancer‐specific aptamers via a random forest model across three cancer types. A) Cancer diagnostic probabilities for different pairwise comparisons using a multivalent aptamer corona. B) ROC‐AUC values for different pairwise comparisons using multivalent aptamer corona. C) Confusion matrix showing the classification performance for each pairwise comparison. D) Network visualization of the 23 aptamers across three cancer types. Color intensity of aptamer nodes denotes their importance in different cancers. E) UMAP visualization shows distinct clustering of OV, LC, and CRC samples based on aptamer binding profiles, highlighting the panel's utility in discerning cancer‐type‐specific signatures. F) Chord diagram illustrating the specific up‐ or down‐regulation of the 23 aptamers across the three cancer types. The ribbon connections integrate the distinct expression patterns that characterize the multi‐cancer diagnostic panel.

To validate the discriminative power of ProteoFish‐SELEX‐evolved multiplexed aptamer corona, we employed a random forest model, partitioning the dataset into training (60%) and testing (40%) subsets. The model demonstrated high inter‐cancer classification accuracy in the testing cohort, with distinct probability distributions differentiating cancer types (**Figure** **5**A). ROC‐AUC analysis further quantified diagnostic precision, yielding high AUC values (0.96‐1.00), including maximal discrimination (AUC = 1.00) between OV and healthy control, as well as OV versus LC (Figure 5B). Confusion matrix analysis of pairwise comparisons corroborated these findings, revealing high sensitivity (>95%) and specificity (>93%) across all cancer subtypes (Figure 5C). This multi‐cancer validation highlights the capacity of multivalent aptamer corona evolved via ProteoFish‐SELEX to serve as precise discriminators in complex clinical matrices, bridging proteomic biomarker discovery to aptamer‐based diagnostic insights.

To delineate aptamer‐cancer associations, we prioritized aptamer candidates that met the following two criteria: i) random forest importance scores >0.3 and ii) top 15 differentially expressed aptamers (DEAs) per cancer type. This yielded a 23‐aptamer panel, including 8 for OV, 7 for LC, and 8 for CRC. Relational network analysis identified 9 aptamers connecting all three malignancies (Figure 5D). Sequence‐specific dominance emerged, with seq42 exhibiting the highest importance in OV and LC, and seq40 in CRC. In addition, the aptamer panel containing 23 cancer‐specific sequences clearly separated the OV, LC, and CRC into 3 different clusters (Figure 5E). Notably, seq42 and seq43 were universally upregulated in malignancies, while seq63, seq60, and seven others were consistently downregulated (Figure 5F). These bidirectional expression trends amplified oncogenic signatures coupled with suppressed regulatory motifs, emphasizing the dual utility of aptamers as positive and negative biomarker sensors for multi‐cancer classification. Overall, ProteoFish‐SELEX‐evolved multivalent aptamer corona identified cancer‐specific aptamers and exhibited excellent performance in high‐throughput multiplexed multi‐cancer diagnosis.

### 2.6 Identification of Cancer‐Specific Aptamer Targeting Proteins Within Aptamer Corona

While aptamers exhibited high specificity in discriminating cancer types, the discovery of their target proteins, particularly those functionally implicated in oncogenic pathways, is essential to unravel their role in cancer pathogenesis.^[^ ^49^, ^50^ ^]^ To identify aptamer‐targeted proteins, we prioritized the diagnostically relevant candidates and focused on the upregulated aptamers due to their potential as positive cancer indicators. Typically, a biotinylated candidate aptamer was mixed with other aptamers and incubated with cancer serum‐derived NPCs to form a protein‐aptamer corona. Then, protein‐aptamer corona was chemically stabilized via formaldehyde fixation prior to buffer‐mediated lysis. Streptavidin bead‐based pulldown was applied to isolate aptamer‐protein conjugates, followed by LC‐MS/MS target protein identification (Figure S19A, Supporting Information). After obtaining the aptamer targeting proteins, corresponding recombinant proteins were purchased for surface plasmon resonance (SPR) binding affinity characterization.

The LC‐MS/MS and SPR profiling revealed that serum amyloid A2 (SAA2) is the target for aptamer OV10. Similar validation resolved cancer‐specific aptamer‐protein pairs are OV2‐C1QB, OV7‐FGB, and OV8‐HRG for ovarian cancer and CRC4‐APOC3, CRC5‐CFHR1, CRC7‐PROS1, CRC8‐C4B, and CRC9‐C8B for colorectal cancer, as well as LC1‐FN1, LC3‐FGA, and LC4‐SERPINC1 for lung cancer. The binding affinity of the above aptamers was listed in Figure S19B–M (Supporting Information). Secondary structure of these identified aptamers was computationally modeled using NUPACK at minimum △G (Figures S20–S22, Supporting Information). Overall, we established a robust pipeline integrating aptamer pulldown, proteomic profiling, and affinity validation to illustrate the aptamer‐protein targeting across ovarian, colorectal, and lung cancers. The evolution of multivalent aptamer corona through ProteoFish‐SELEX demonstrated scalable screening across protein abundance tiers, enabling discovery of both high‐ and low‐abundance cancer‐associated proteins as annotated in Figure 3I–K.

## 3 Conclusion

Aptamers hold immense potential for biomarker detection and precision therapeutics owing to their high binding affinity, stability, and adaptability.^[^ ^51^, ^52^ ^]^ However, the rapid enzymatic degradation of aptamers in serum or plasma significantly hinders their translational application in blood‐based in vitro diagnosis. We developed a multivalent aptamer corona strategy via ProteoFish‐SELEX, a serum‐based aptamer selection platform leveraging NPC technology. By adsorbing thousands of serum proteins onto magnetic nanoparticles, NPC preserves native molecular complexity while shielding aptamer libraries from enzymatic degradation. With cancer‐associated proteins and cancer‐specific aptamers, a multivalent aptamer corona achieved a precise multiplexed multi‐cancer diagnosis in high throughput.

Proteomic profiling of NPCs derived from 292 clinical serum samples identified 1338 serum proteins and revealed cancer‐associated proteins in OV, LC, and CRC. These findings confirm that NPCs retain disease‐specific epitopes, allowing detection via multivalent aptamer corona evolved by ProteoFish‐SELEX. Using this approach, we generated 27, 30, and 40 cancer‐specific aptamers for OV, LC, and CRC, respectively, which effectively discriminated cancer patients from healthy controls in an independent validation cohort. A critical step in validating our platform was the implementation of a rigorous blinded diagnostic assessment using an independent clinical cohort (*n* = 142). By combining DEAs from different cancer types, we established a refined panel containing 69 unique aptamers after deduplication. Aptomic profiling was performed using 142 aptamer coronas, with a reference sequence incorporated for normalization. A random forest model, trained on 60% of this cohort and subjected to a strict blind test on the held‐out 40%, demonstrated exceptional diagnostic accuracy, with AUC values ranging from 0.96 to 1.00 for discriminating cancers from healthy controls as well as differentiating cancer types. This robust validation underscores the clinical potential of our approach. Furthermore, to elucidate the molecular basis of aptamer binding, we identified 12 high‐affinity aptamer‐protein pairs. It is important to clarify that these pairs were primarily characterized to verify specificity and binding affinity. The diagnostic model itself, however, was built upon a broader, synergistically acting 23‐aptamer panel identified through feature importance analysis. Notably, the panel comprised both upregulated and downregulated aptamers, highlighting their dual utility as positive and negative biomarkers in a combinatorial diagnostic strategy. This multi‐directional profiling capability enhances the versatility of the platform for pan‐cancer stratification and underscores its clinical translational potential.

The performance of our platform underscores a clear advantage over aptamer discovery approaches dependent on cultured cells, such as conventional cell‐SELEX. Although cell‐SELEX has been valuable for selecting aptamers against membrane proteins of specific cell types, its reliance on cultured cells restricts its access to the broader pathophysiological proteome present in vivo. In contrast, the ProteoFish‐SELEX strategy directly interrogates the complex protein milieu of patient serum. This approach not only enables the discovery of aptamers against a wider range of biomarkers, including low‐abundance secreted proteins, but also preserves the inherent natural characteristics of proteins in clinical samples. The successful identification of diagnostic aptamer panels and their high‐affinity protein targets from serum cohorts, rather than from predefined cellular models, underscores the methodological advancement of our platform for liquid biopsy applications.

While this study advances critical groundwork for aptamer‐based diagnostics, its translation into routine clinical practice faces several challenges. First, pooled serum aliquots were employed in ProteoFish‐SELEX to enhance molecular diversity, the clinical cohort size remains modest. Scaling to larger, multi‐center cohorts with diverse cancer subtypes and stages is critical to validate biomarker generalizability and advance translation research. Second, equimolar pooling of prioritized aptamers for multi‐cancer discrimination introduces potential bias, as their pathophysiological relevance may not align with molar stoichiometry. Third, quantifying the functional weight of individual aptamers in a pooled assay poses significant technical challenges, necessitating innovative normalization strategies in future work. Furthermore, iterative screening yielded only 12 high‐affinity aptamers, partially due to structural discrepancies between recombinant proteins and native target proteins within NPCs. Finally, subsequent studies should incorporate benign controls and evaluate the platform's performance across various disease stages, including metastatic settings, which is crucial for fully establishing its real‐world clinical utility.

In summary, our multivalent aptamer corona represents a paradigm shift in aptamer‐based in vitro diagnosis for complex biological samples. Aptamer corona evolution via ProteoFish‐SELEX facilitates high‐throughput screening directly from clinical serum cohorts, eliminating risks associated with enzymatic degradation and obviating the requirement for purified recombinant protein targets during SELEX. Through validation across multi‐cancer serum cohorts via high‐throughput multiplexed target protein detection with high accuracy, multivalent aptamer corona demonstrates its capacity and potential as a versatile tool for broad biomarker discovery and detection.

## 4 Experimental Section

### Materials

For the synthesis of nanoparticles, the ferric chloride hexahydrate (FeCl3·6H2O) was purchased from Sigma‐Aldrich. Trisodium citrate, sodium acetate (NaAc), tetraethyl orthosilicate (TEOS), ammonium hydroxide (NH3·H2O), and ethanol were purchased from Aladdin (Shanghai, China). All the chemicals mentioned above were of analytical grade. Highly pure water with an electrical resistivity of 18.2 MΩ·cm^−1^ was used throughout all experiments. For LC‐MS/MS sample preparation, Trizma hydrochloride (reagent, ≥ 99%), DL‐dithiothreitol (for molecular biology, ≥ 99% HPLC), iodoacetamide (IAA), acetonitrile (gradient grade, ≥ 99.9%) and sodium chloride (NaCl) were purchased from Sigma‐Aldrich, and Pierce C18 Tips (87784), trifluoroacetic acid (TFA), and formic acid (FA) was purchased from Thermo Fisher Scientific. For protein SELEX, a random ssDNA library, primers, and biotin‐labeled primers were purchased from Shanghai Sangon Biotech (China). BSA and tRNA were purchased from Solarbio (China). TaKaRa Taq (R001B) was purchased from Takara. Streptavidin Sepharose High Performance affinity resin (17‐5113‐01) was purchased from Cytiva (GE Healthcare Life Science). The complement C1q subcomponent subunit B (C1QB) was purchased from Sino Biological (China), the fibrinogen beta chain (FGB) and fibrinogen alpha chain (FGA) were purchased from AbbVie (England). Serum amyloid A‐2 protein (SAA2), complement factor H‐related protein 1 (CFHR1), and apolipoprotein C‐III (APOC3) were purchased from Novoprotein (Shanghai, China; CR50, C585, DRA275). The complement C4 (C4B) and complement component C8 beta chain (C8B) were purchased from Cloud‐Clone. The histidine‐rich glycoprotein (HRG), fibronectin (FN1), vitamin K‐dependent protein S (PROS1), and antithrombin‐III (SERPINC1) were purchased from MCE.

### Recruitment of Clinical Cohort Samples

Serum samples were collected from patients with different types of cancer and from healthy volunteers. The groups were categorized as follows: Healthy (*n* = 200), Ovarian (*n* = 187), NSCLC (*n* = 214), and Colorectal (*n* = 100). The samples were obtained from Zhejiang Cancer Hospital (ZJCH). The study was approved by the Ethics Committee of ZJCH and complied with all relevant ethical regulations (Ovarian cancer: IRB‐2021‐416; NSCLC: IRB‐2024‐664; Colorectal cancer: IRB‐2022‐350). Prior to serum sample collection, all participating patients and healthy volunteers were fully informed of the study's purpose, procedures, potential risks, and rights. Written informed consent was obtained from each participant in accordance with ethical guidelines and regulatory requirements. Clinical characteristics, including age, sex, cancer type, and stage, are summarized in Table S1 and Table S2 (Supporting Information).

### Nanoparticle‐Protein Corona Formation and Proteolytic Digestion

The desired NPs were incubated with human serum at a 1:1 volume ratio for 1 h under constant agitation to form a nanoparticle‐protein corona. After incubation, the nanoparticle‐protein corona was separated from serum using magnetic separation and washed with 150 mM NaCl to remove unbound corona proteins. To digest the corona proteins, NPCs were resuspended in Tris‐HCl buffer containing 10 mm dithiothreitol at 95 °C for 10 min. Proteins were denatured with urea for 30 min. Subsequently, proteins were treated with 20 mM iodoacetamide (IAA) for 30 min in the dark. Proteins were then hydrolyzed with trypsin (Promega, Madison, WI, USA) overnight at an enzyme‐to‐substrate ratio of 1:50 in 100 mM ammonium acetate. A 10% trifluoroacetic acid (TFA) solution was used to deactivate the trypsin. Peptides were desalted using Pierce C18 Pipette Tips according to the manufacturer's instructions, followed by vacuum drying. The peptides were stored at −80 °C for further LC‐MS/MS analysis.

### ProteoFish‐SELEX Procedures

ProteoFish‐SELEX was performed by incubating the DNA library with NPCs, followed by elution of the bound DNA, which was then amplified by PCR. To mitigate inter‐individual variability, 6 serum samples were randomly selected from 6 different subjects and pooled into a single aliquot. This strategy was scaled into six parallel aliquots, each containing pooled samples from six unique cancer patients (total *n* = 36) for one single positive‐SELEX pipeline. For complementary counter‐SELEX, an equivalent cohort of healthy donors (*n* = 36), underwent identical pooling to eliminate nonspecific binders. This pooled design ensured the generation of broadly generalizable aptamers robust against biological heterogeneity. The ProteoFish‐SELEX workflow commenced with a negative selection round using sera from healthy subjects. NPCs were formed by incubating MNPs with serum, followed by buffer washes to remove unbound proteins. A single‐stranded DNA library with 66 nucleotides (18‐nt primers flanking a 30‐nt randomized region) was then introduced into NPCs, enabling the formation of aptamer corona through sequence‐target binding. The unbound sequences from six parallel negative selection workflows were pooled and advanced to the positive‐SELEX phase. For positive selection, NPCs were reconstituted using sera from cancer patients, preserving disease‐specific proteomic complexity. Aptamer candidates surviving counter‐SELEX were incubated with cancer‐derived NPCs to form a target‐specific aptamer corona. Nonbinding sequences were removed through stringent washing, while high‐affinity aptamers bound to target proteins were recovered via elution. Six, or the ideal number of, cycles of negative‐ and positive‐SELEX were performed, after which the final enriched aptamer pool from the last positive selection round underwent next‐generation sequencing to identify candidate sequences. To assess the capacity of ProteoFish‐SELEX for identifying cancer‐specific biomarkers and their cognate aptamers, the platform was applied to clinical serum cohorts encompassing ovarian cancer (OV), lung cancer (LC), and colorectal cancer (CRC), alongside healthy donor controls.

Typically, for OV serum‐based ProteoFish‐SELEX, counter‐SELEX commenced by incubating 50 µL of MNPs (1 mg) with 50 µL of serum from healthy donors for 1 h to form stable nanoparticle‐protein coronas (NPC). NPCs were washed with 300 µL NaCl (150 mM) three times to remove the unbound proteins and resuspended in a solution containing 300 µL of ssDNA library (OD = 1.0). The ssDNA library was heated at 95 °C for 10 min and then rapidly cooled on ice for 10 min to allow the DNA library to form a stable secondary structure before incubation with NPCs. Aptamer corona was formed after incubation for 1 h at room temperature. Then, unbound DNA library sequences were collected from the six parallel counter‐SELEX and mixed into one tube for positive‐SELEX. Similarly, in a typical positive‐SELEX, 50 µL of MNPs (1 mg) were incubated with 50 µL of serum from OV patients for 1 h to form stable NPCs. Unbound proteins were washed away using a NaCl solution. Then, NPCs were resuspended in an unbound DNA library from counter‐SELEX (300 µL) for 1 h to form an aptamer corona. Aptamer corona was washed using DPBS buffer three times. Bound aptamers were eluted using DNase‐free water by heating for 10 min at 95 °C, followed by magnetic separation to collect the supernatant for qPCR detection. The eluted aptamers were then used as a template for PCR amplification with the forward primer and biotin‐labeled reverse primer. The PCR products were captured using streptavidin Sepharose high‐performance beads, and the fluorophore‐labeled amplicons were subsequently isolated from the biotinylated antisense DNA by elution with 100 mm NaOH. Six ssDNA pools were collected and used for the next cycle of counter‐SELEX and positive‐SELEX. After six or seven cycles, eluted aptamers of the last positive SELEX from the six parallels were pooled into one tube before advancing to next‐generation sequencing. Detailed information was summarized in Table S4 (Supporting Information). For LC and CRC, ProteoFish‐SELEX was conducted under the same conditions.

### Clinical Cohort Sample Discrimination Validation Using Enrichment Pools from the Last Positive‐Selex

The enrichment pools (ssDNA) of the last positive‐SELEX from each cancer type were amplified by PCR. The PCR products were separated by streptavidin Sepharose high‐performance beads, and the fluorophore‐labeled amplicons were isolated from the biotinylated antisense DNA through elution with 100 mm NaOH. In a typical discrimination validation (OV vs healthy control as an example), 50 pmol of ssDNA were incubated with NPCs derived from OV serum samples (*n* = 19) and healthy control serum samples (*n* = 19) for 1 h at room temperature to form stable aptamer coronas. The aptamer coronas (*n* = 38) were washed three times to remove nonspecifically bound aptamers. Then, 200 µL of DNase‐free water was added to each sample and heated at 95 °C for 10 min to elute the ssDNA bound to corona proteins. After elution, the ssDNA was amplified by PCR, and the PCR products were isolated using streptavidin Sepharose high‐performance beads. To ensure sequencing fidelity, a standardized reference sequence (TGGATGTGTGTGTTGTGTAGTTGCTCTGAT) was spiked into each eluted sample prior to next‐generation sequencing. Normalization against this internal standard enabled robust cross‐sample quantification. Similarly, independent discrimination validation on LC and CRC was conducted using the sample protocol (LC: *n* = 30; CRC: *n* = 30).

### Clinical Cohort Sample Discrimination Validation Using Synthetic Differentially Expressed Aptamers from the Enrichment Pools

Differentially expressed aptamers were obtained from the discrimination validation of OV, LC, and CRC clinical cohorts. The differentially expressed aptamers were thus synthesized in each cancer type, including 27 in ovarian cancer, 30 in lung cancer, and 40 in colorectal cancer. A new independent clinical cohort was used for cancer discrimination validation. Typically (OV vs healthy control as an example), the 27 synthesized differentially expressed aptamers (OV: *n* = 27) were mixed in a 1:1 molar ratio. Fifty pmol of synthetic aptamer mixture were incubated with NPCs derived from OV serum samples (*n* = 14) and healthy control serum samples (*n* = 14) for 1 h at room temperature to form stable aptamer coronas. The aptamer coronas (*n* = 28) were washed three times to remove nonspecifically bound aptamers. Then, 200 µL of DNase‐free water was added to each sample and heated at 95 °C for 10 min to elute the ssDNA bound to corona proteins. After elution, the ssDNA was amplified by PCR, and the PCR products were isolated using streptavidin Sepharose high‐performance beads. To ensure sequencing fidelity, a standardized reference sequence (TGGATGTGTGTGTTGTGTAGTTGCTCTGAT) was spiked into each eluted sample prior to next‐generation sequencing. Normalization against this internal standard enabled robust cross‐sample quantification. Similarly, discrimination validation using 30 (LC) and 40 (CRC) differentially expressed aptamers was conducted separately using the sample protocol with independent clinical cohorts (LC: *n* = 48; CRC: *n* = 48).

### Multiple Cancer Discrimination Validation Using All Differentially Expressed Aptamers Synthesized Enriched Pools for Detection in Multiple Cancers

To evaluate the multi‐cancer diagnostic utility of aptamers, a composite aptamer panel was curated comprising sequences differentially expressed in the final positive SELEX for OV (27), LC (30), and CRC (40). After deduplication, a refined panel of 69 unique aptamers was synthesized and pooled at equimolar ratios for downstream validation (Table S9, Supporting Information). The 69 unique aptamers were synthesized that were differentially expressed in OV, LC, and CRC, and mixed them in a 1:1 molar ratio to create a composite library. This panel was then tested against an expanded independent clinical cohort (*n* = 142), including 36 OV, 35 LC, 35 CRC, and 36 healthy subjects, with demographic and staging metadata detailed in Table S2 (Supporting Information). Typically, 50 pmol of synthetic aptamer mixture was incubated with NPCs derived from OV, LC, CRC, and healthy control serum for 1 h at room temperature to form stable aptamer coronas. The aptamer coronas (*n* = 142) were washed three times to remove nonspecifically bound aptamers. Then, 200 µL of DNase‐free water was added to each sample and heated at 95 °C for 10 min to elute the ssDNA bound to corona proteins. After elution, the ssDNA was amplified by PCR, and the PCR products were isolated using streptavidin Sepharose high‐performance beads. To ensure sequencing fidelity, the same standardized reference sequence (TGGATGTGTGTGTTGTGTAGTTGCTCTGAT) was spiked into each eluted sample prior to next‐generation sequencing. Normalization against this internal standard enabled robust cross‐sample quantification. The sequencing data were analyzed as described above.

### LC‐MS/MS for Identification of Target Proteins

To identify the target proteins for each cancer‐associated aptamer, the upregulated aptamers were selected in each cancer type. These aptamers were synthesized with 5’‐end biotin modifications, while the remaining aptamers were unmodified. For instance, to identify OV2 aptamer‐targeted protein, the OV2 aptamer was modified with a biotin group. Then, it was mixed with the other 26 regular aptamers without biotin modification to form a composite pool. The resulting aptamer mixture was incubated with NPCs derived from OV serum and healthy control serum separately to form an aptamer corona. After 1 h of incubation, a formaldehyde solution (final concentration: 2%) was introduced to the aptamer corona and incubated for 30 min to crosslink the aptamers and proteins. Subsequently, an ammonium bicarbonate solution was added to quench the formaldehyde crosslinking reaction for 15 min. The aptamer‐protein complexes were lysed using lysis buffer and incubated with 200 µg of streptavidin‐modified magnetic beads for 10 min at room temperature. After incubation, the beads were washed three times with DPBS and resuspended in Tris‐HCl for LC‐MS/MS sample preparation. This process was described previously in proteolytic digestion.

### Binding Kinetics Measurement Using SPR

The binding affinity Kd value of target aptamers was determined by SPR assay on a Biacore‐8K instrument with CM5 chips at ambient temperature (25 °C). The detailed aptamer sequences were listed in Table S10 (Supporting Information). Briefly, 3 µg of recombinant human target protein were immobilized on a CM5 chip for binding experiments with candidate aptamers and a His‐tagged protein as a control. DPBS solution containing 5 mM Mg^2^⁺ was used as the running buffer. Aptamers (150 µL) were injected into the Biacore‐8K at the following series of concentrations: 0, 62.5, 125, 250, 500, and 1000 nm. After a 180‐s contact time, dissociation was allowed for 180 s at a flow rate of 30 µL min^−1^. Surface regeneration was performed using 1.5 M NaCl solution. Binding data were analyzed with Biacore Insight Evaluation Software.

### Statistical Analysis

R or Python was used for proteomics and aptomics data analysis. Missing values of the protein group in the MS data were imputed using the median. GraphPad Prism 9 (GraphPad Software, USA) was utilized for statistical analysis and data visualization. Statistical analysis was performed with one‐way analysis of variance (ANOVA) for comparisons among differentially expressed proteins or aptamers across different cancers. Results were considered statistically significant if the corrected *p*‐value was <0.05. Characterization data were derived from the mean values of three independent experiments. Data were expressed as mean ± SD and sample size (*n*) for each statistical analysis is represented in the corresponding figure legends.

## Conflict of Interest

The authors declare no conflict of interest.

## Supporting information



Supporting Information

Supporting Information

## Data Availability

The data that support the findings of this study are available in the supplementary material of this article.
