# Supplementary material for: Evolution of Multivalent Aptamer Corona for High‐Throughput Multiplexed Detection of Multiple Cancers
Source: Adv Sci (Weinh). 2025 Nov 25;13(8):e14976. doi: 10.1002/advs.202514976 (PMC12884786; doi:10.1002/advs.202514976)
Supplement: Supplementary file 1 — Supporting Information [file ADVS-13-e14976-s002.docx]

Supporting Information

**Evolution of Multivalent Aptamer Corona for High-Throughput Multiplexed Detection of Multiple Cancers**

Mengjie Wang, Baichuan Jin, Xin Dai, Haozhe Huang, Qiqi Liu, Jianqing Zhu, Haixing Ju, Qixun Chen, Yongmei Song, Yuan Liu*, Weihong Tan*

**Experimental Methods**

**Synthesis of Fe_3_O_4_@SiO_2_ NPs.** Synthesis of MNPs was performed according to previously published methods.^[1]^ First, the iron oxide core was synthesized by dissolving FeCl_3_·6H_2_O (2.8 g) in ethylene glycol under sonication. Afterward, NaAc (4.20 g) and trisodium citrate (1.0 g) were added, and the mixture was stirred vigorously. The mixture was transferred to a sealed Teflon-lined stainless-steel autoclave (100 mL capacity). The autoclave was heated to 200 °C for 10 h and then cooled to room temperature. Finally, the product was washed with deionized water and ethanol. Subsequently, a silica shell was formed around the Fe₃O₄@SiO₂ NP core. Typically, 0.5 g Fe₃O₄ NPs were dispersed in 10 mL of deionized water and added dropwise to 400 mL of ethanol under sonication for 30 min. Next, 10 mL of aqueous ammonia solution and 3 mL of TEOS were added, followed by stirring at 600 rpm for 3.5 h. Finally, MNPs were collected by magnetic separation and washed three times with deionized water and ethanol.

**Characterization of MNPs and nanoparticle-protein corona.** Size and zeta potential were measured using dynamic light scattering (DLS; Zetasizer Nano ZS, ZEN3600, Malvern, UK). NPs were dispersed in deionized water (10-50 mg/mL) and sonicated prior to measurement. DLS measurements were conducted using disposable polystyrene semi-micro cuvettes. Zeta potential was measured using disposable folded capillary cells (Malvern Instruments, DTS1070) after a 1 min equilibration time at 25 °C. Size and morphology were additionally analyzed by transmission electron microscopy (TEM) using a JEM-F200 microscope (JEOL, Japan) at 200 kV. For TEM analysis, samples were dispersed in ethanol or deionized water and then deposited onto carbon-film-coated copper grids.

**Data-independent acquisition (DIA) of LC-MS/MS.** The lyophilized peptide was reconstituted in 0.1% formic acid, and 500 ng of peptide from each sample were analyzed using a Thermo Scientific Easy-nLC 1200 system coupled to an Orbitrap Exploris 480 mass spectrometer (Thermo Fisher Scientific). Peptides were loaded onto an Acclaim PepMap C18 trapping column (75 μm × 25 cm) and separated on an analytical column at a flow rate of 350 nL/min using a gradient of 3%-45% solvent B (80% acetonitrile, ACN) over 62 min, followed by 45%-95% solvent B for 2 min and maintained for 10 min, for a total run time of 80 min. The mass spectrometer was operated in full width at half maximum (FWHM) resolution mode using 30 variable windows across the 400-2000 *m/z* range. Prior to LC-MS/MS analysis of samples, a quality control sample (Thermo Fisher Scientific) was run to assess instrument performance.

**MS data analysis.** DIA raw data files were processed and analyzed using Spectronaut (*version 18*) with default settings. MS raw files were searched against the human UniProt FASTA database (UP000005640, 74,349 forward entries; downloaded in August 2019). A library-based search was employed to analyze the raw data. DIA data were analyzed using our previously established deeply fractionated ProteoFish spectral library.^[2]^ Sample information, including protein type, intensity, gene name, and molecular weight (MW), was exported as a CSV file.

The CSV files were analyzed using R software, generating heatmaps, principal component analysis (PCA), volcano plots, upset plots, and Gene Ontology (GO) and Kyoto Encyclopedia of Genes and Genomes (KEGG) pathway enrichment analyses. Differential protein expression was evaluated using the R packages ggplot2 and limma, with a significance threshold of *p* < 0.05. Differentially expressed proteins in each group were visualized using volcano plots for each cancer type. Enrichment analysis of upregulated and downregulated proteins was conducted using the clusterProfiler package, and significant enrichment was identified with a cutoff threshold of *p* < 0.05. For each sample, pathway activity scores were calculated as the average expression levels of differentially expressed genes associated with the enriched pathway. Classification models were constructed to differentiate between cancer and healthy samples, with a 60:40 training-to-testing split. Receiver operating characteristic (ROC) analysis was performed to assess the sensitivity and specificity of the classification and summarized by area under the curve (AUC) scores.

**DNA library and primers.** The initial ssDNA library consisted of a randomized 30-nucleotide region flanked by two primer binding sites: 5’-CAGCACCGTCAACTGAAT-N30-GTGATGCGATGGAGATGT-3’. The forward primer and the reverse primer were labeled with FAM and biotin, respectively. The forward primer was 5’-FAM-CAGCACCGTCAACTGAAT-3’, and the reverse primer was 5’-biotin-ACATCTCCATCGCATCAC-3’. The detailed sequence information is shown in Table S3.

**High-throughput sequencing of DNA aptamers from ProteoFish-SELEX.** The eluted ssDNA pools of the last positive SELEX from different cancer types were sent to Sangon Biotech (Shanghai, China) for high-throughput sequencing analysis using an Illumina platform. Paired-end sequencing was performed on NovaSeq 6000/MiSeq sequencers with PE150/PE300 models (Illumina, San Diego, CA). The sequencing results were downloaded using FileZilla software. Subsequent clinical validation cohort samples were sent to Sangon Biotech for high-throughput sequencing analysis.

**Sequencing data analysis.** Sequencing data from different cancer types were analyzed using R software. Principal component analysis (PCA) was used to reduce data dimensionality, and differential aptamer expression was assessed using the ggplot2 and limma R packages, with a significance threshold of *p* < 0.05. Upregulated and downregulated differentially expressed aptamers in each group were visualized using volcano plots. Sequences that were differentially expressed between healthy control and cancer samples in each enrichment pool were selected for further analysis. Specific sequence information of the differentially expressed aptamers in each cancer type is provided in Table S5-S7.

**Data Dependent Acquisition (DDA) of LC-MS/MS.** LC-MS/MS analysis was performed on a Thermo Fisher Scientific Easy-nLC 1200 system coupled to an Orbitrap Exploris 480 mass spectrometer. The mobile phases consisted of 0.1% formic acid in water (phase A) and 0.1% formic acid in acetonitrile (phase B) with the flow rate set to 350 nL/min. The gradient was run over 65 minutes: starting with 3% solvent B (80% acetonitrile, 0.1% formic acid) for 1 minute, followed by an increase to 8% solvent B over 1 minute, then from 8% to 32% solvent B over 57 minutes, from 32% to 100% solvent B over 2 minutes, finally maintaining 100% solvent B for 5 minutes. The mass spectrometer was operated in data-dependent acquisition (DDA) mode, covering an *m/z* range of 400-2000.

The raw data files were processed using Proteome Discoverer (PD) software (Thermo Fisher Scientific). The MS raw data were searched against the *Homo sapiens* subset of the human UniProt FASTA database (UP000005640; 74,349 forward entries, version downloaded August 2019). Carbamidomethylation of cysteine was specified as a fixed modification. The precursor mass tolerance and fragment mass tolerance were set to 10 ppm and 0.02 Da, respectively. Peptide and protein reports were exported as CSV files, and subsequent statistical and visualization analyses were performed using custom R scripts.

1. **Supplementary Figures**


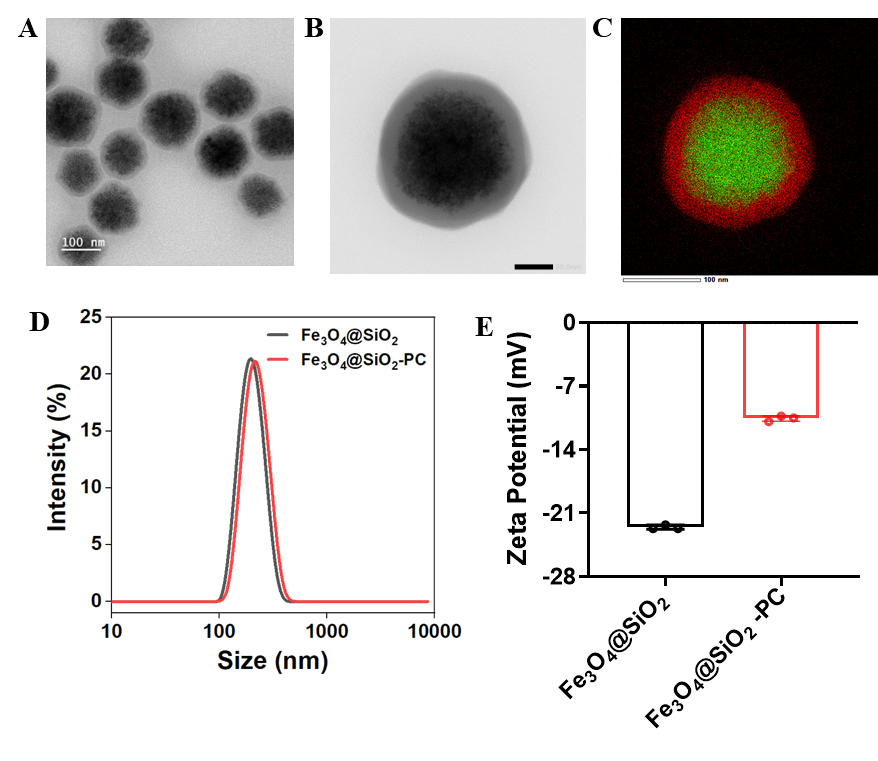


**Figure S1.** Characterization of Fe_3_O_4_@SiO_2_ NPs and nanoparticle-protein corona. (A, D, E) HRTEM images of Fe_3_O_4_@SiO_2_ NPs. (B, C) Size and surface charge of Fe_3_O_4_@SiO_2_ NPs before and after formation of nanoparticle-protein corona. Mean ± S.D., n = 3. (F) Images of Fe_3_O_4_@SiO_2_ NPs elemental mapping for Fe (green) and Si (red).

**
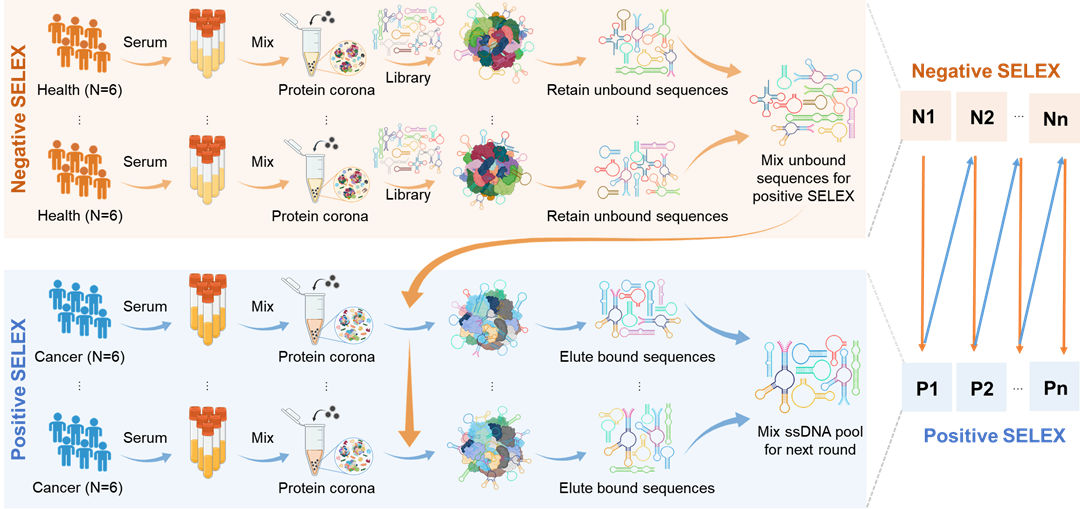
**

**Figure S2.** Workflow of ProteoFish-SELEX.

**
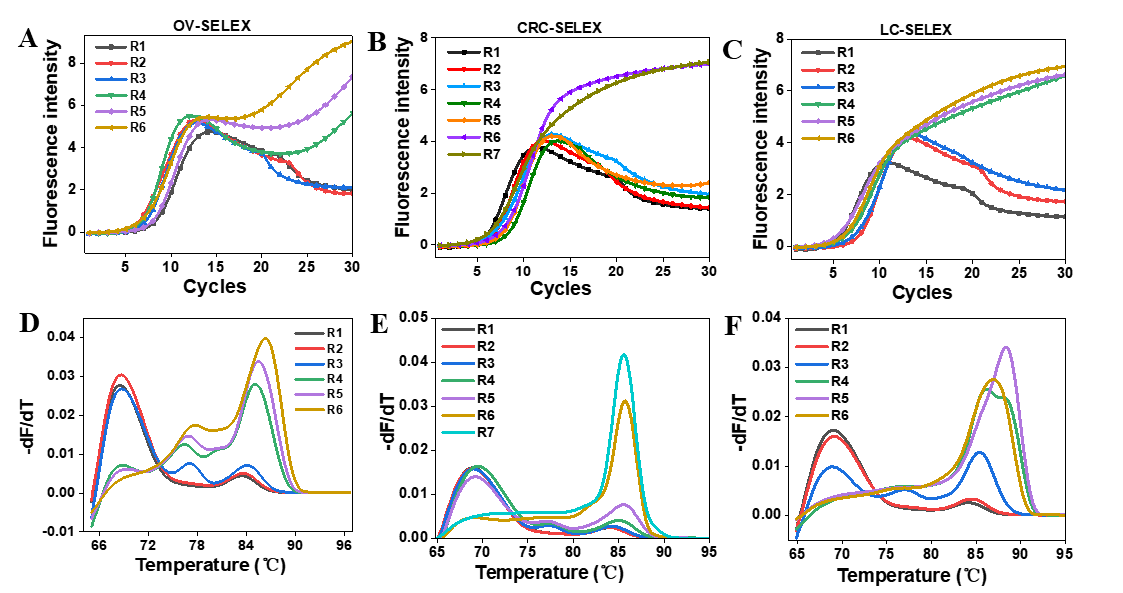
**

**Figure S3.** Amplification curves and melting curves of eluted ssDNA from each round of positive ProteoFish-SELEX. (A, D) OV selection. (B, E) CRC selection. (C, F) LC selection.


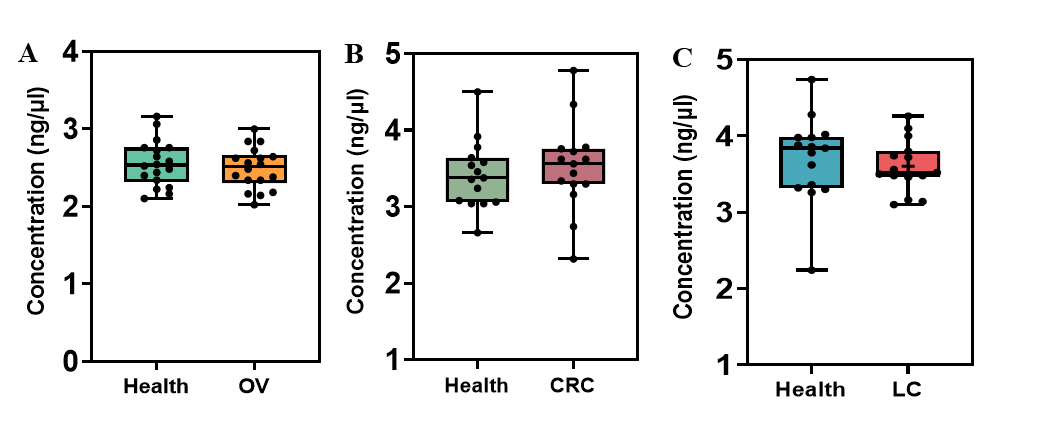


**Figure S4.** Quantitative analysis of aptamers eluted from NPCs isolated from serum samples of cancer patients and healthy controls. (A) OV (n=48), (B) CRC (n=30), (C) LC (n=30).


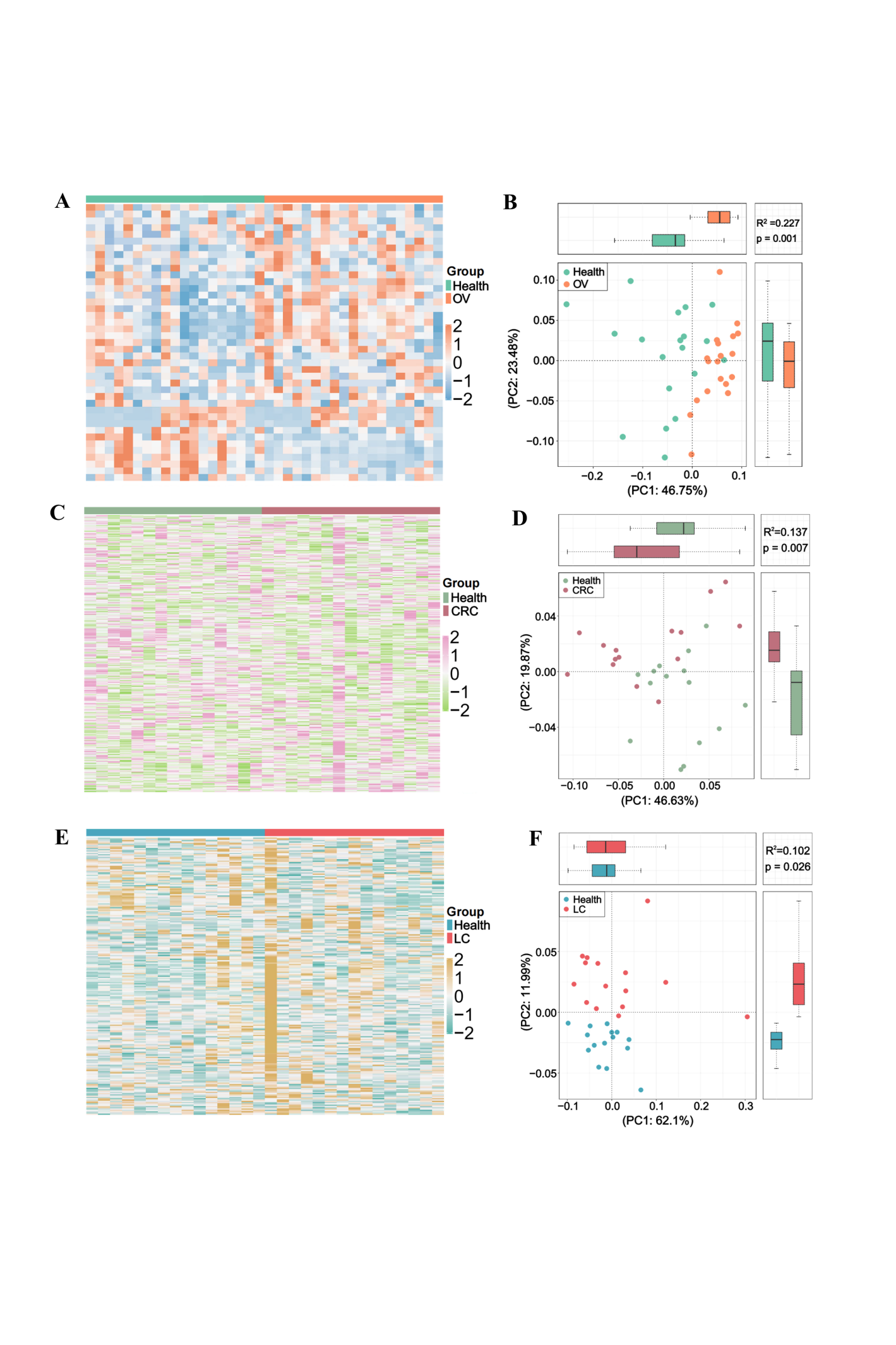


**Figure S5.** Analysis of eluted aptamers from cancer discrimination validation. (A, C, E) Heatmap indicating the expression level of eluted aptamers from NPCs derived from cancer patient serum and healthy control serum. (A) OV, (C) CRC, (E) LC. (B, D, F) PCA plot analysis of clinical serum cohort validation using the last round enriched pools from OV (B), CRC (D), and LC (F), respectively.


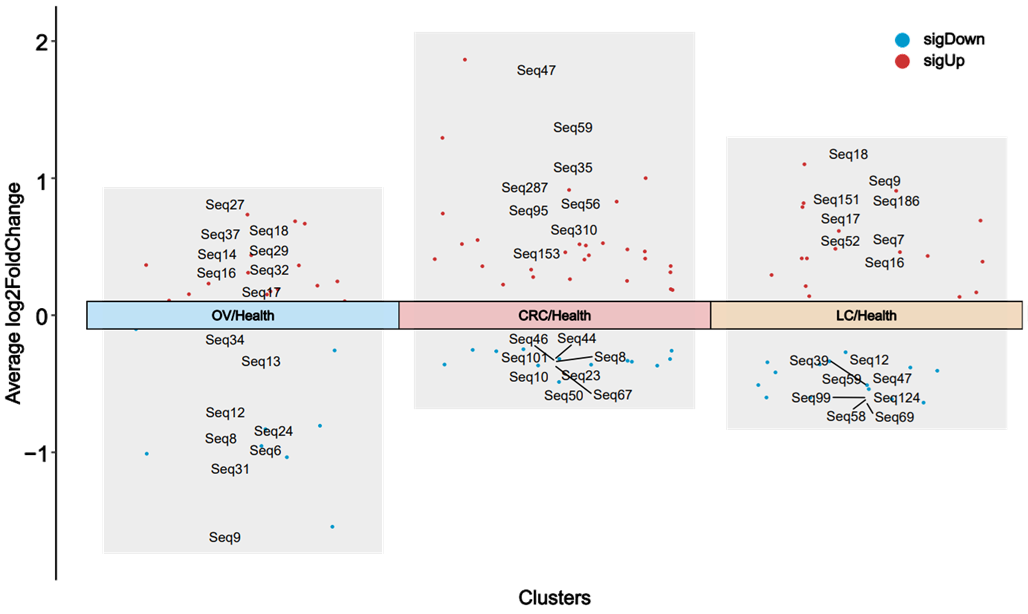


**Figure S6.** Volcano plot of differentially expressed aptamers in different cancer types using enriched pools.


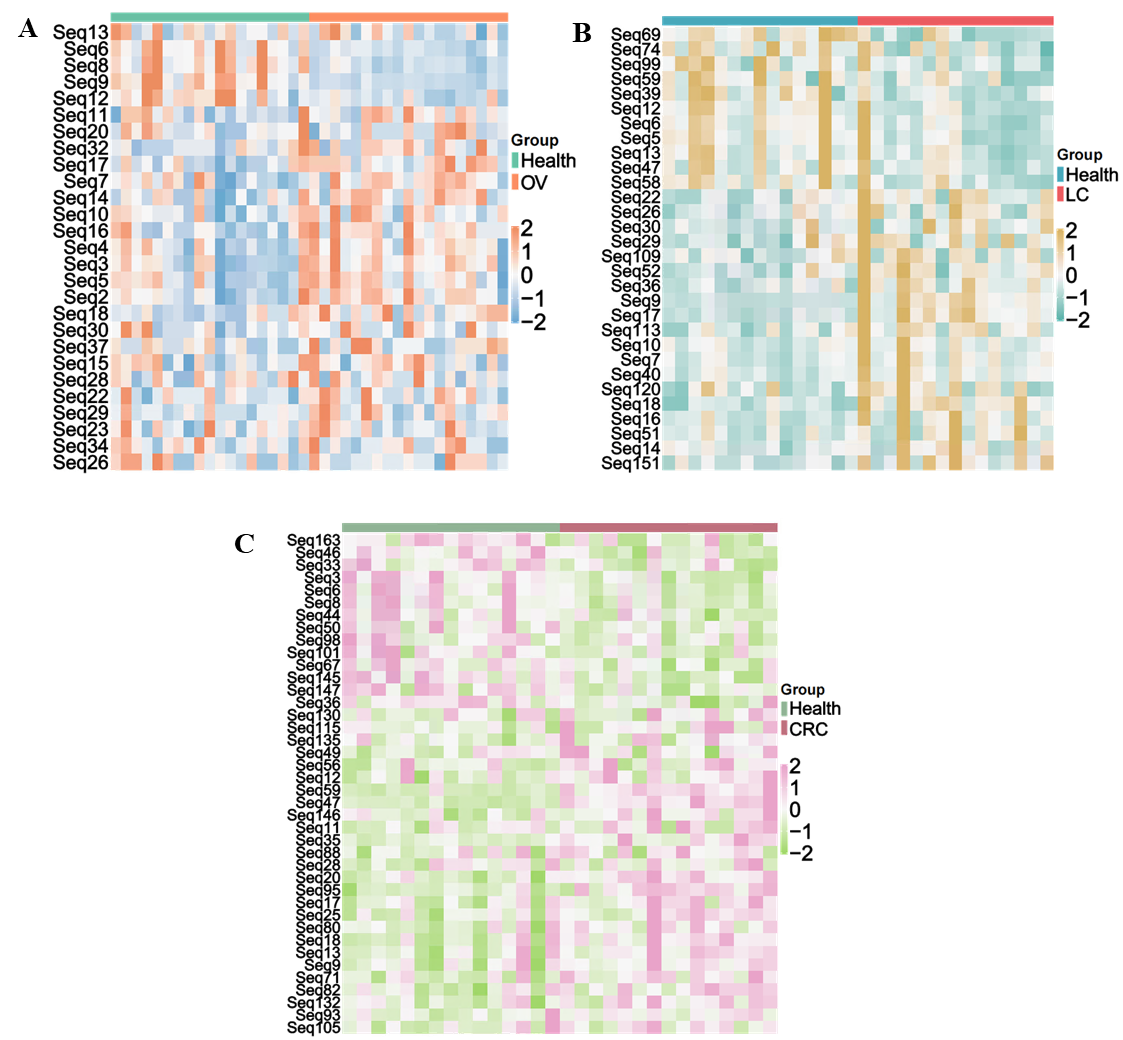


**Figure S7.** Discrimination analysis of differentially expressed aptamers using enriched pool in distinct cancers (Enriched pools were amplified through PCR). (A) Heatmap of 27 differentially expressed aptamer sequences in OV (n = 38). (B) Heatmap of 30 differentially expressed aptamer sequences in LC (n = 30). (C) Heatmap of 40 differentially expressed aptamer sequences in CRC (n = 30).


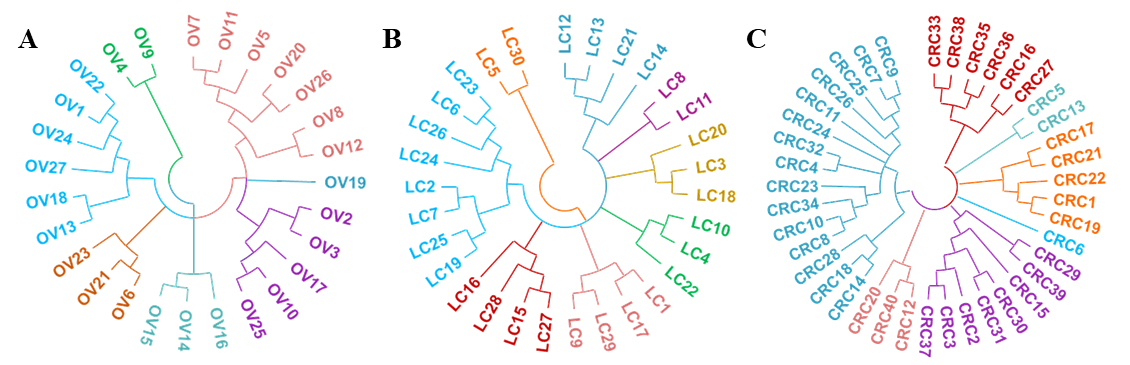


**Figure S8.** Phylogenetic reconstruction of differentially expressed aptamers using MEGA software. A-C The phylogenetic tree analysis of the 27 differentially expressed aptamer sequences in OV (A), 30 differentially expressed aptamer sequences in LC (B), and 40 differentially expressed aptamer sequences in CRC (C).


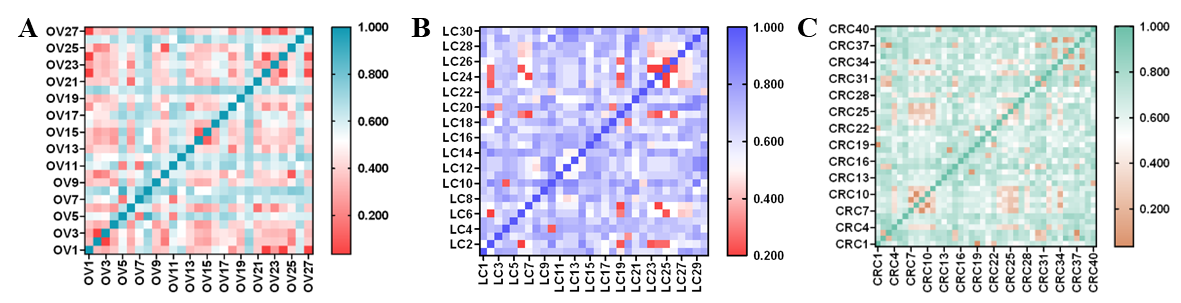


**Figure S9.** Pearson correlation analysis of differentially expressed aptamers using MEGA software. (A-C) Pearson correlations of the 27 differentially expressed aptamers in OV (A), 30 differentially expressed aptamers in LC (B), 40 differentially expressed aptamers in CRC (C).

**
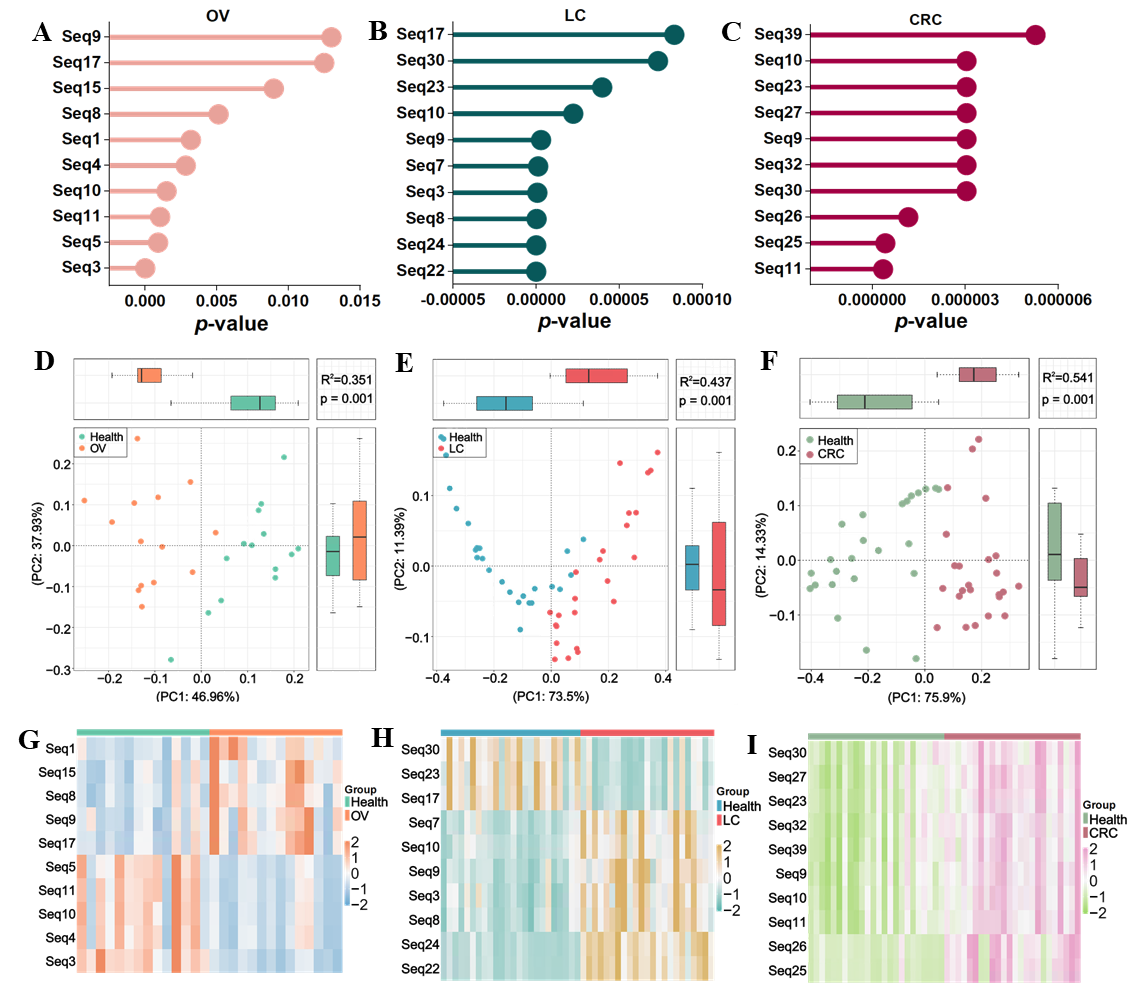
**

**Figure S10.** Top 10 aptamer refinement from synthetic differentially expressed aptamers and discrimination validation in different cancers. (A-C) Top 10 aptamer rank based on p-value importance for OV (A), LC (B), and CRC (C). The y-axis denotes aptamers. The x-axis denotes p-value. (D-F) PCA plots using the top 10 aptamer panels for OV (D), LC (E), and CRC (F). (G-I) Heatmaps of the selected top 10 aptamers ranked by p-value importance generated with three independent synthetic aptamer pools, using external independent serum cohorts from OV (G), CRC (H) and LC (I), respectively.


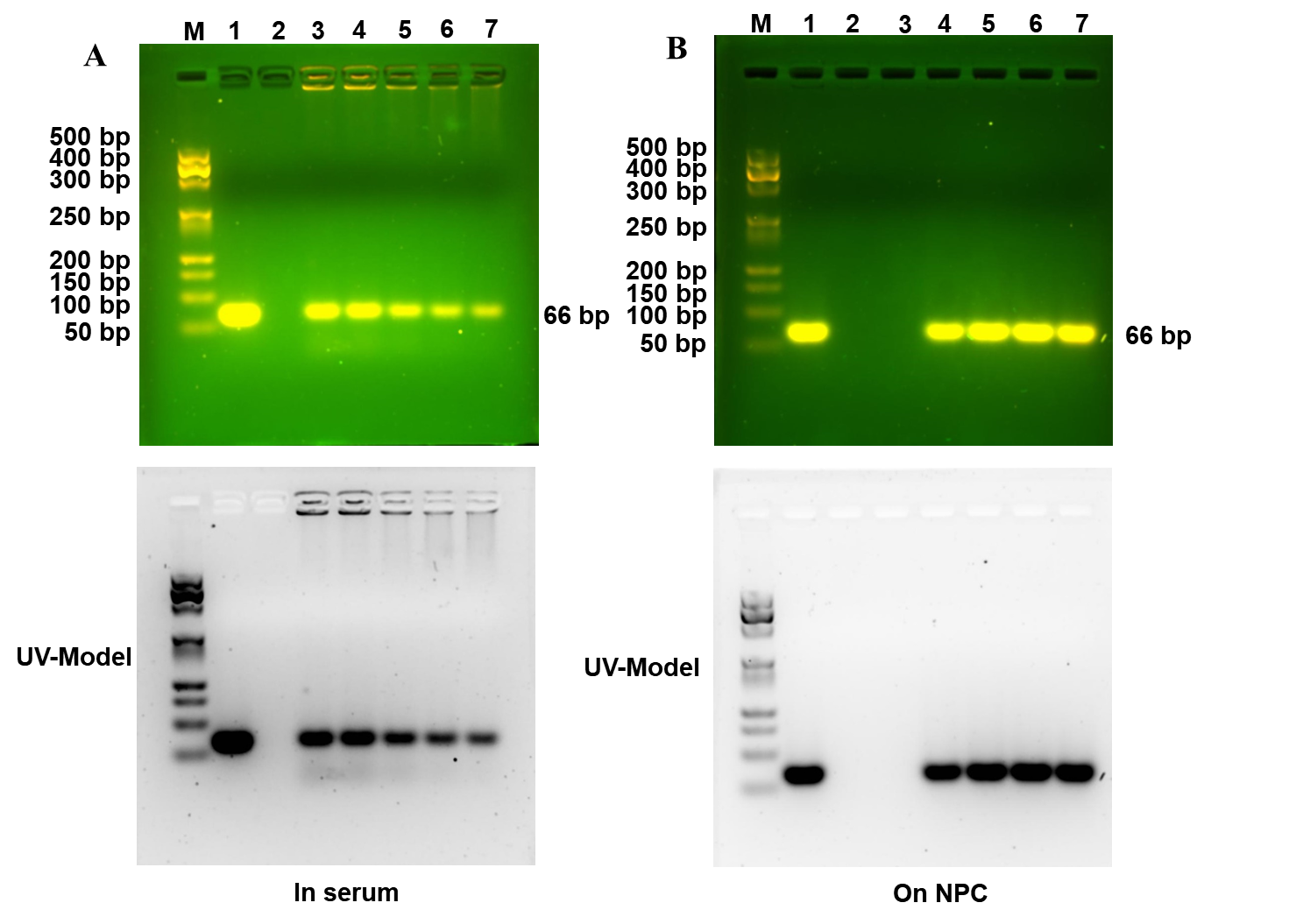


**Figure S11.** Analysis of aptamer (Seq2 in OV) degradation after incubation with serum and NPC over time. Degradation was assessed using agarose gel electrophoresis and visualized under both fluorescence (FL) and UV modes. (A) In serum: lane M: Marker; lane 1: Seq2 aptamer only; lane 2: serum only; Lane 3: Seq2 + serum, 1 min; Lane 4: Seq2 + serum, 10 min; Lane 5: Seq2 + serum, 30 min; Lane 6: Seq2 + serum, 60 min; Lane7: Seq2 + serum, 120 min. (B) On surface of NPC: lane M: Marker; lane 1: Seq2 aptamer only; lane 2: serum only; Lane 3: Seq2 + NPC, 1 min; Lane 4: Seq2 + NPC, 10 min; Lane 5: Seq2 + NPC, 30 min; Lane 6: Seq2 + NPC, 60 min; Lane7: Seq2 + NPC, 120 min.


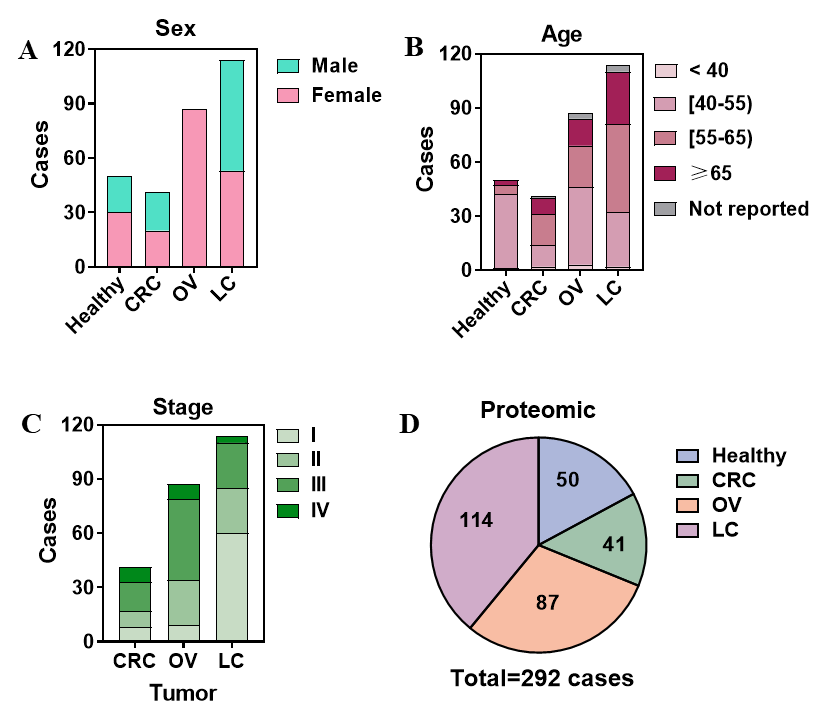


**Figure S12.** Detailed demographic information of the clinical cohorts for proteomic profiling. (A-C) Sex, age, and staging information for proteomic cohort samples. (D) The numbers of different cancer types and healthy control in proteomic profiling (n = 292).


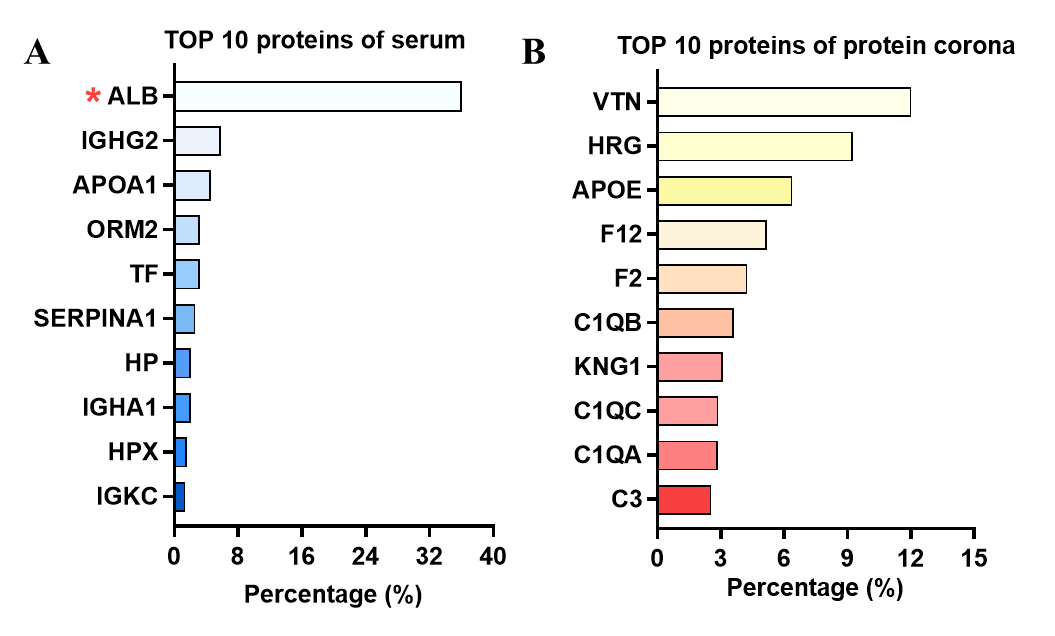


**Figure S13.** Comparison of serum and protein corona. (A-B) The top 10 proteins accounts for 72.89% for serum (A), 52.15% for protein corona (B).


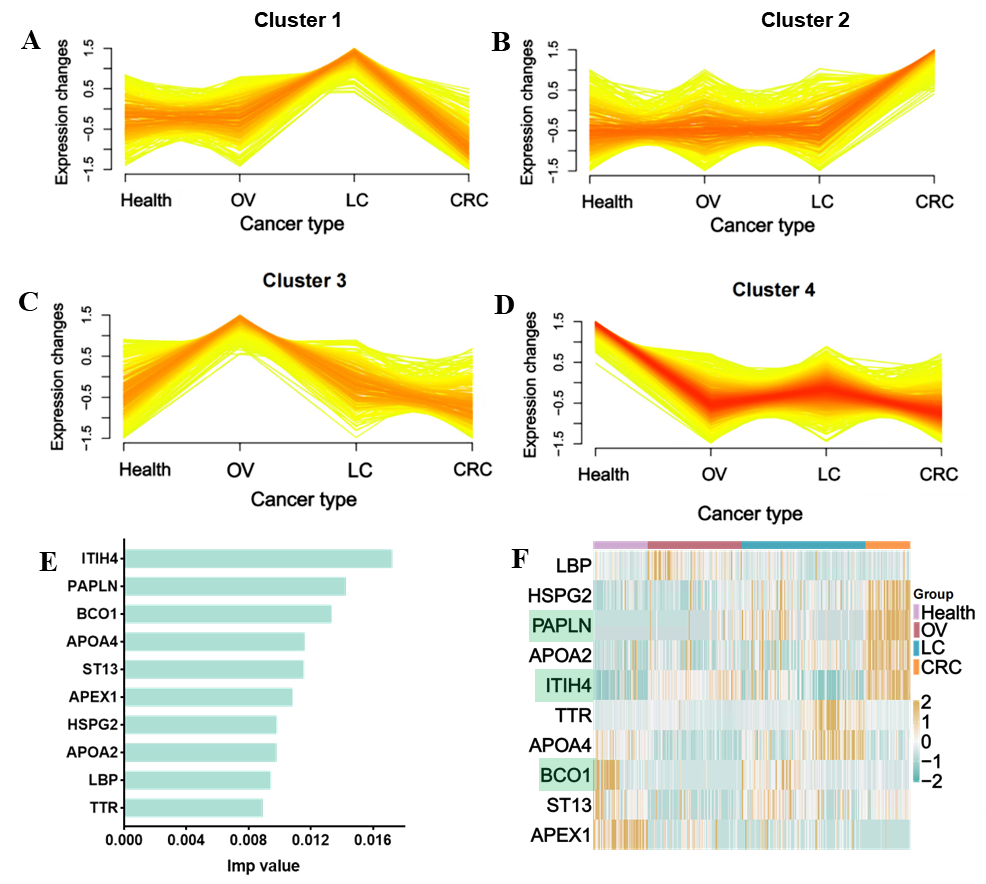


**Figure S14.** K-means clustering of healthy control and multiple cancers. (A-D) Proteins were grouped into four clusters based on the expression similarity in different cancer types. (E) Rank of Top 10 key proteins selected by random forest algorithm based on importance value. (F) Heatmap of the top 10 key proteins showing distinct patterns across the Healthy, OV, LC, and CRC groups. The top three proteins, ranked by importance, are highlighted in green.


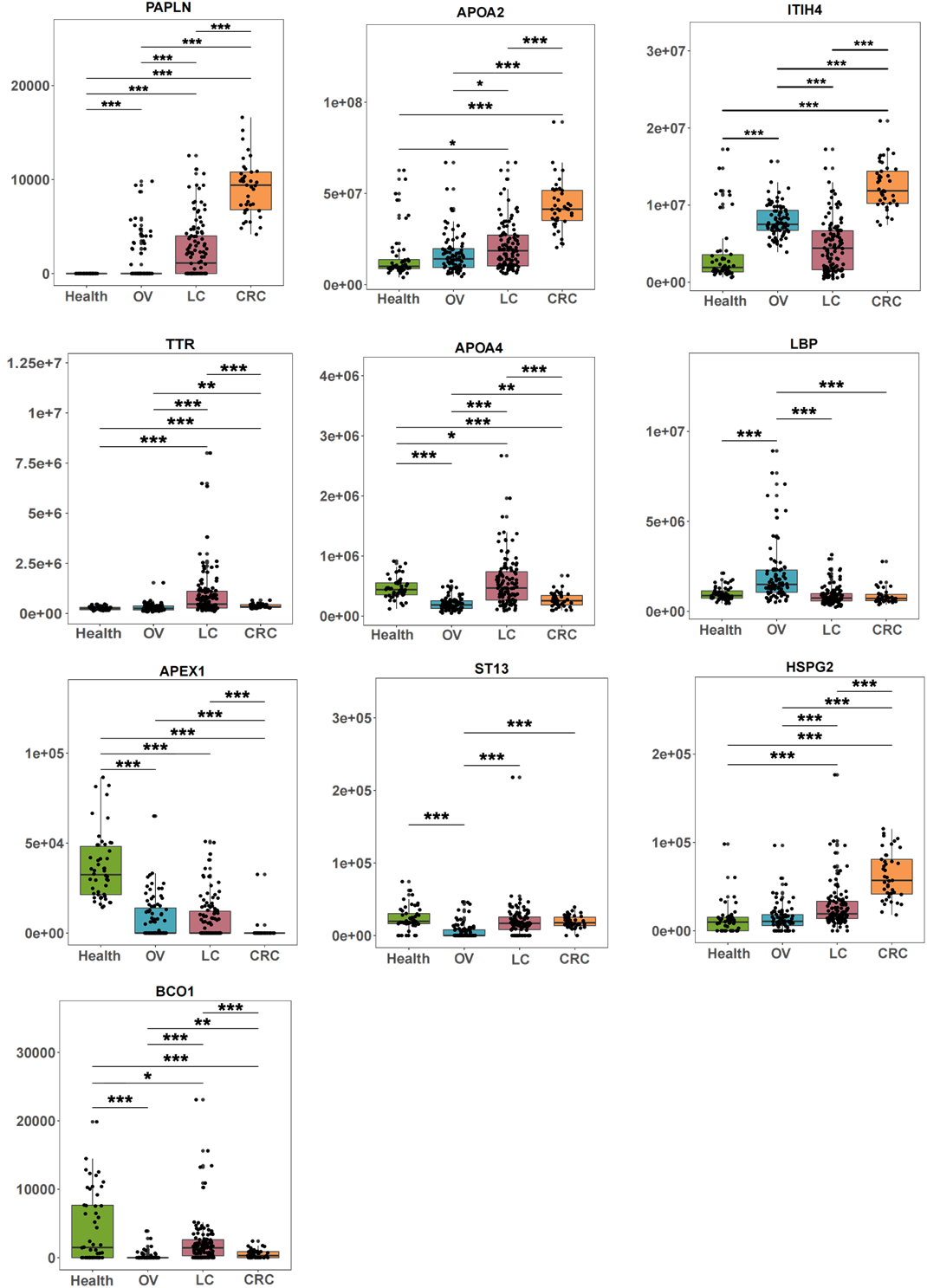


**Figure S15.** Protein levels of the top 10 key proteins selected by random forest model across different cancer types. *P*-value was calculated using one-way ANOVA.


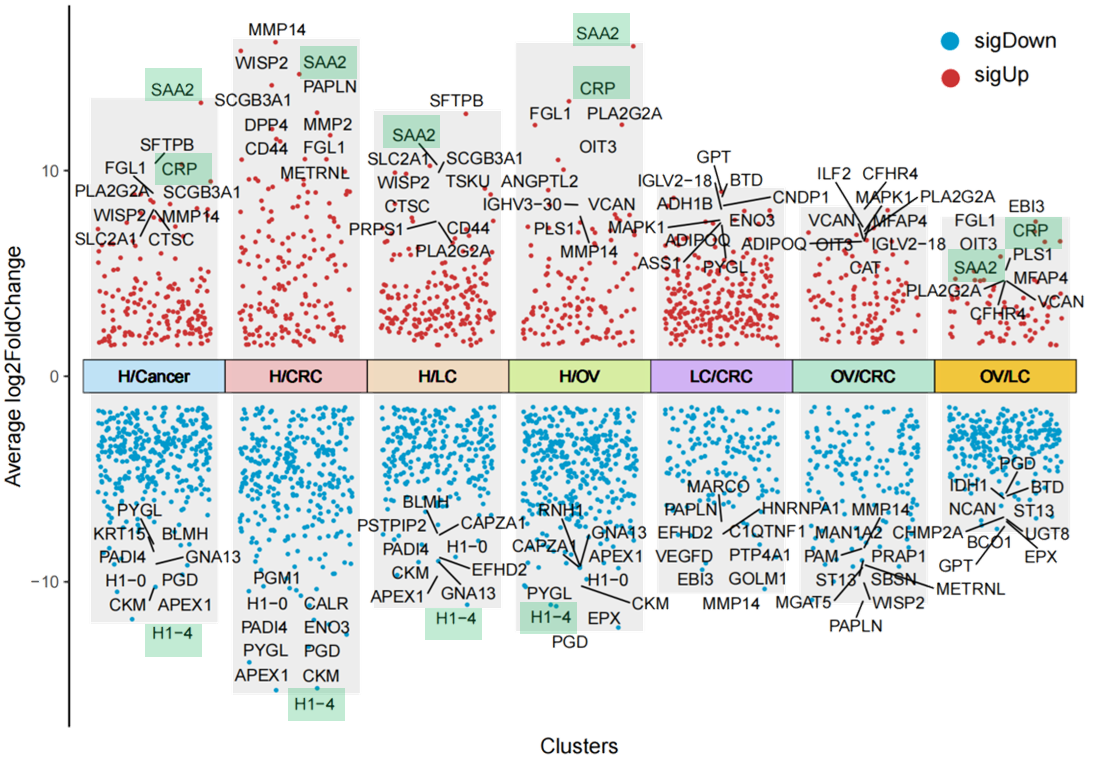


**Figure S16.** Volcano plot of differentially expressed proteins in different comparisons. Proteins highlighted in green represent those with significant up-regulation and down-regulation.


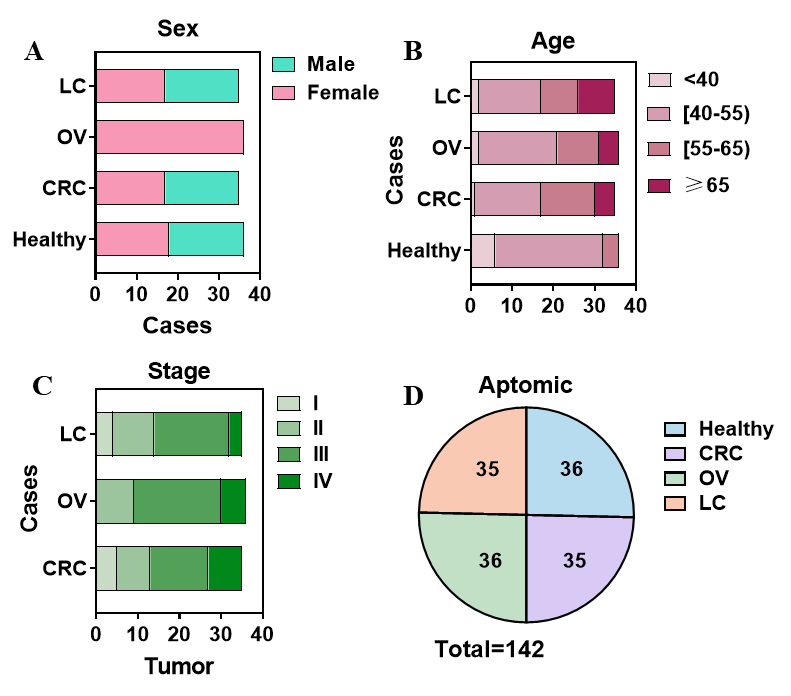


**Figure S17.** Detailed demographic information of the clinical cohorts for aptamer-based multi-cancer discrimination validation. (A-C) Sex, age, and staging information for aptamer-based multi-cancer discrimination validation cohort samples. (D) The numbers of different cancer types and healthy control in aptamer-based multi-cancer discrimination validation (n = 142).


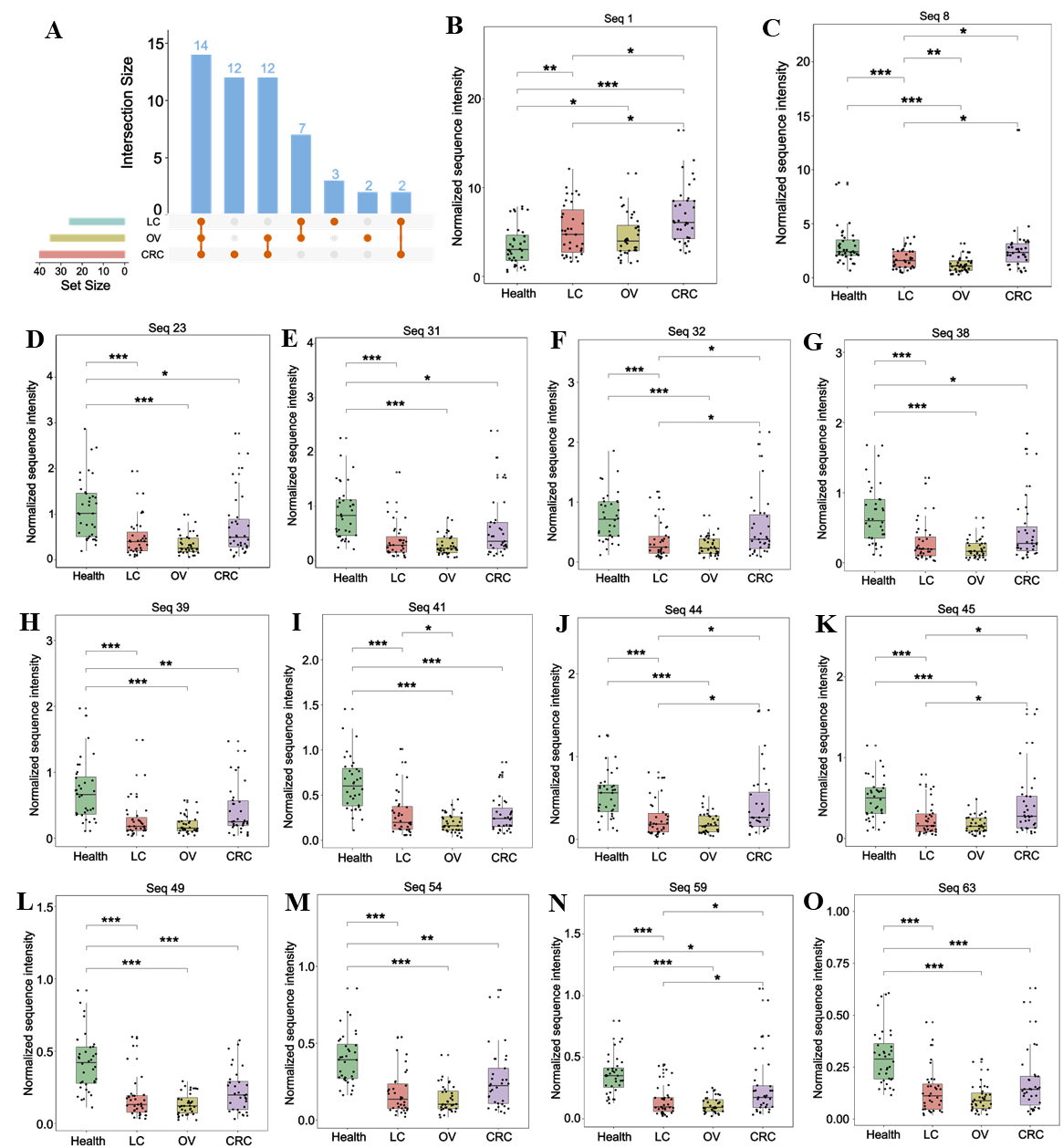


**Figure S18.** Expression level of the 14 common aptamers in different cancers. (A) Upset plot showing the numbers of common and unique differentially expressed aptamers in different cancer types. (B-O) Relative expression levels of the 14 common aptamers (normalization against the internal standard reference sequence was applied) in healthy control, LC, OV, and CRC. *P*-value was calculated using one-way ANOVA.


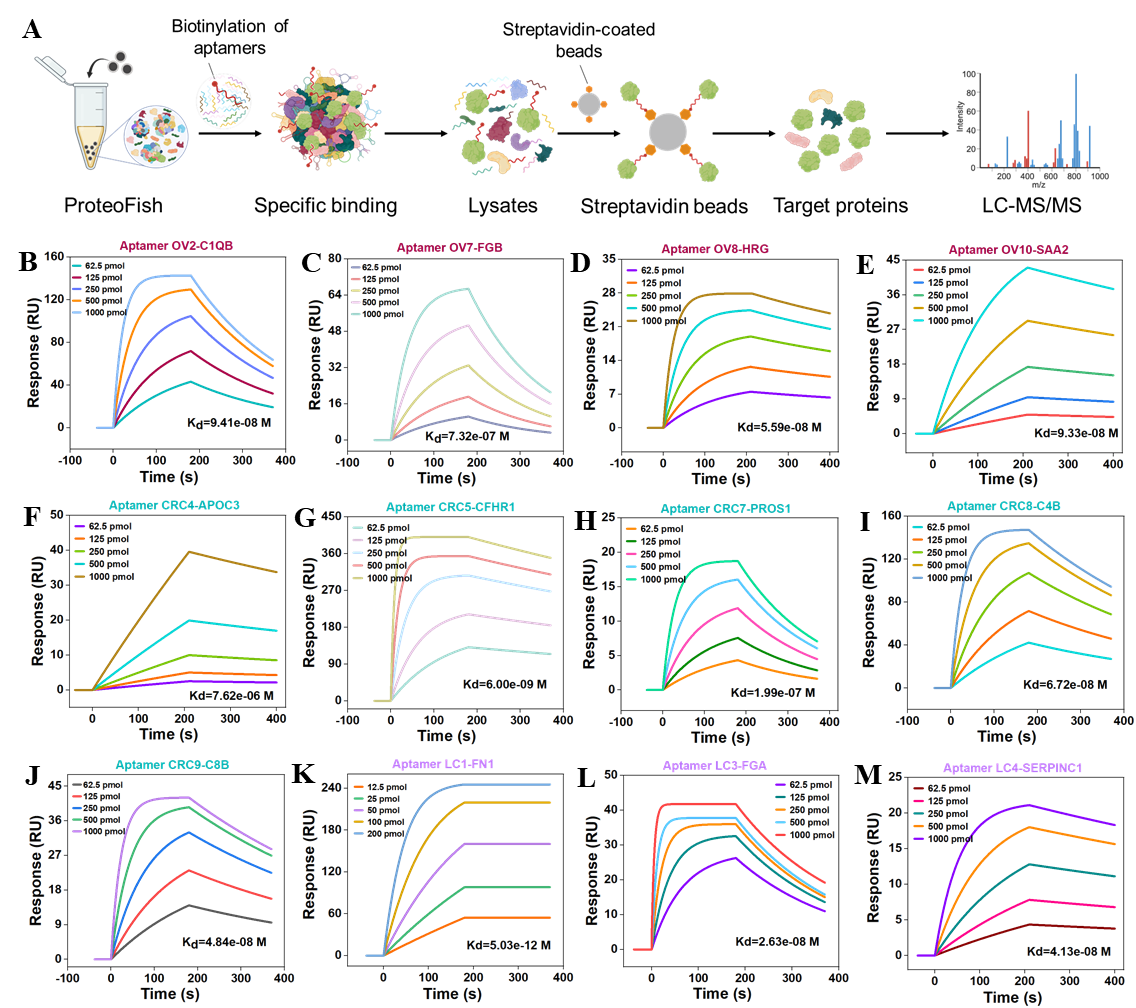


**Figure S19.** Identification and binding affinity characterization of aptamer bound cancer-associated target proteins. (A) Identification workflow for candidate protein biomarkers and aptamers in different cancers. (B-M) SPR analysis showing the binding affinity of aptamers to their corresponding target proteins in OV (B-E), CRC (F-J), and LC (K-M).


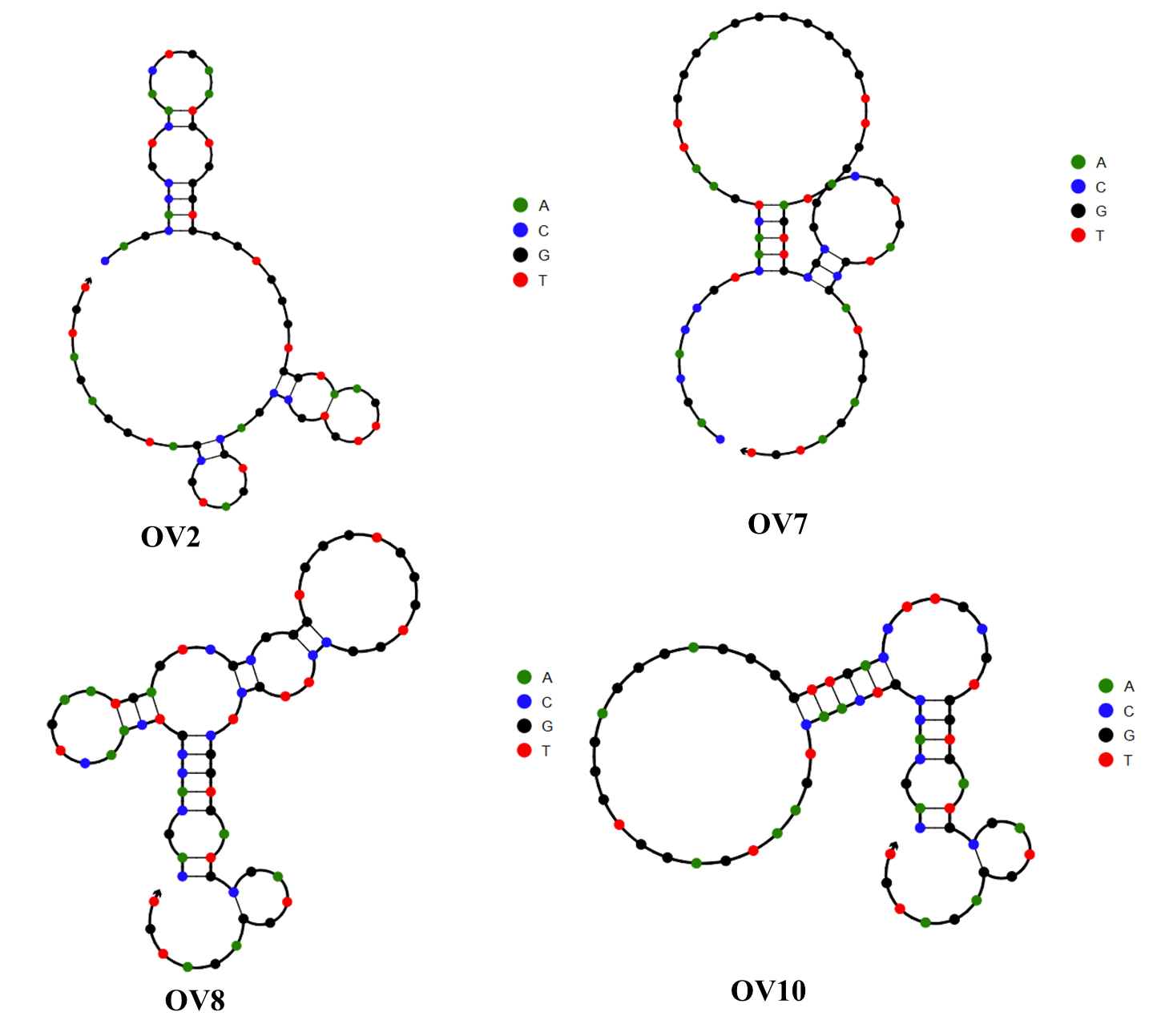


**Figure S20.** Predicted secondary structure of the 4 high-affinity aptamers to OV using NUPACK.


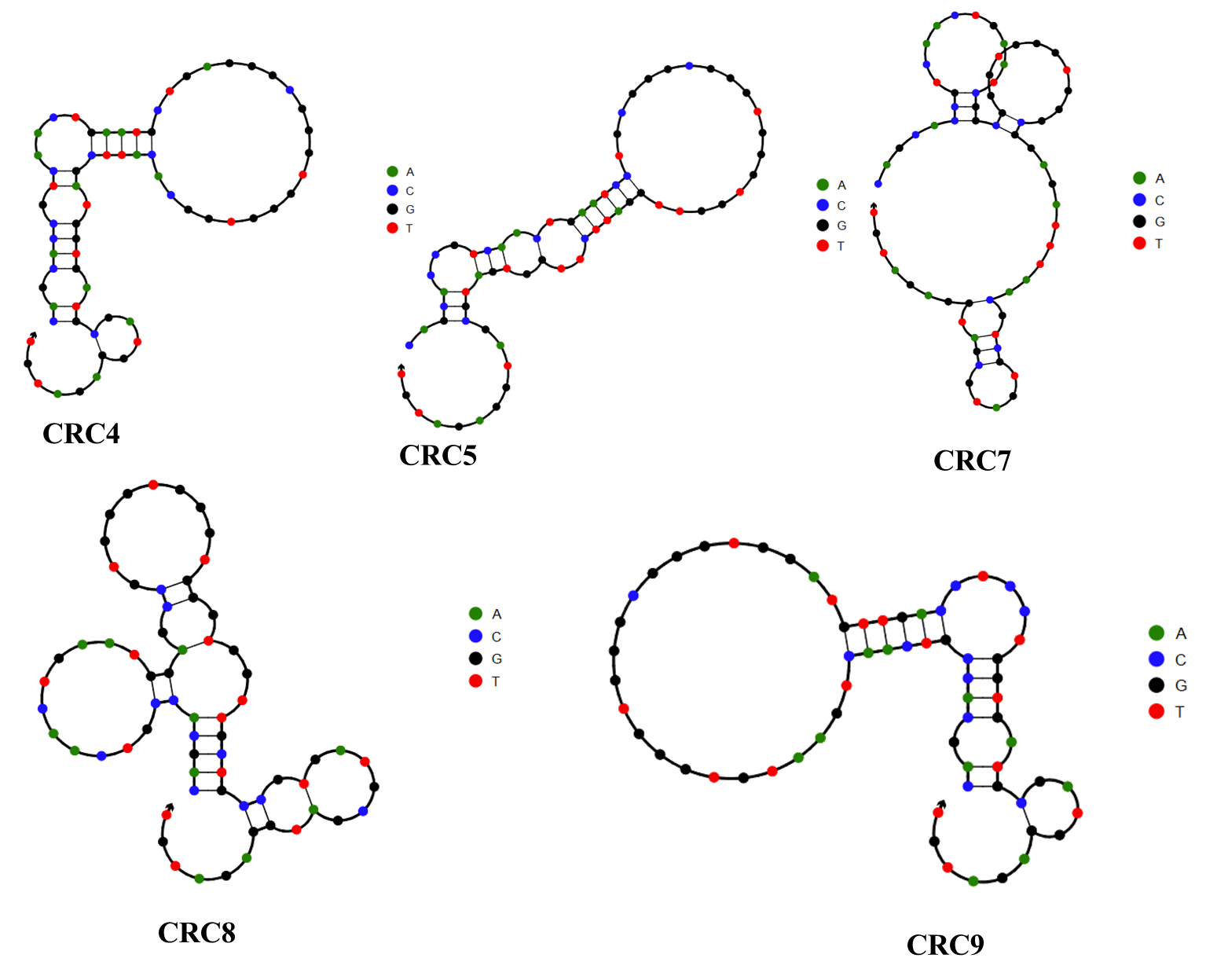


**Figure S21.** Predicted secondary structure of the 5 high-affinity aptamers to CRC using NUPACK.

**
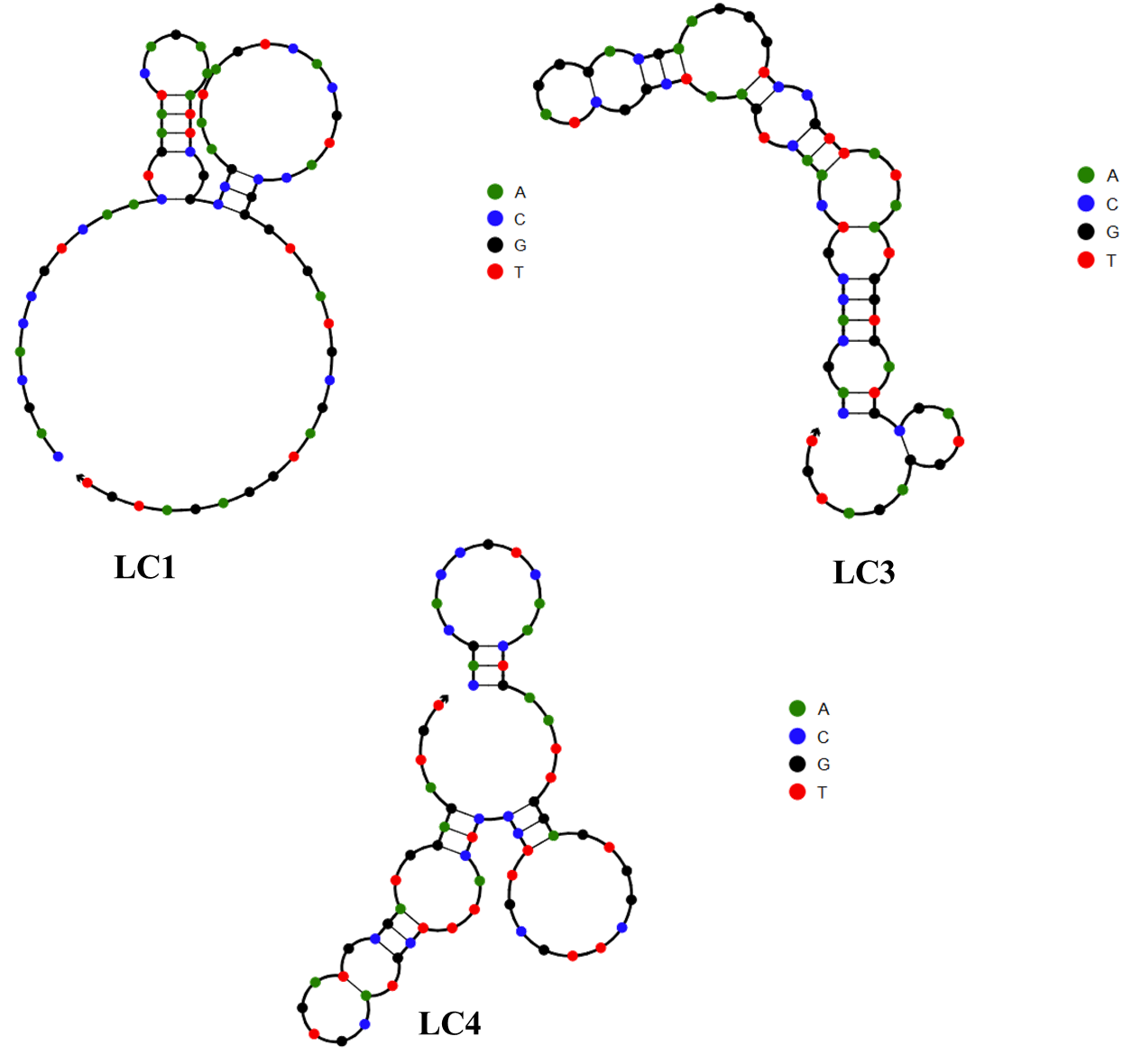
**

**Figure S22.** Predicted secondary structure of the 3 high-affinity aptamers to LC using NUPACK.

1. **Supplementary Table**

**Table S1.** Clinical pathologic characteristics of participants in proteomic profiling cohort (n = 292).

| Variables | | Health  （n = 50） | OV  （n = 87） | LC  （n = 114） | CRC  （n = 41） |
| --- | --- | --- | --- | --- | --- |
| Age at diagnosis | Mean ± SD. | 44±4 | 55±9 | 59±9 | 58±10 |
|  | Range | 39-55 | 33-82 | 33-81 | 35-77 |
| Gender | Males, n | 20 | 0 | 61 | 20 |
|  | Females, n | 30 | 87 | 53 | 21 |
| Pathological  stage | Ⅰ | - | 9 | 60 | 8 |
|  | Ⅱ | - | 25 | 25 | 9 |
|  | Ⅲ | - | 45 | 25 | 16 |
|  | Ⅳ | - | 8 | 4 | 8 |

**Table S2.** Clinical pathologic characteristics of participants in aptamer-based multi-cancer discrimination validation (n = 142).

| Variables | | Health  （n = 36） | OV  （n = 36） | LC  （n = 35） | CRC  （n =35） |
| --- | --- | --- | --- | --- | --- |
| Age at diagnosis | Mean ± SD. | 41±4 | 53±10 | 57±11 | 55±9 |
|  | Range | 35-53 | 33-74 | 37-77 | 38-72 |
| Gender | Males, n | 18 | 0 | 18 | 18 |
|  | Females, n | 18 | 36 | 17 | 17 |
| Pathological  stage | Ⅰ | - | 0 | 4 | 5 |
|  | Ⅱ | - | 9 | 10 | 8 |
|  | Ⅲ | - | 21 | 18 | 14 |
|  | Ⅳ | - | 6 | 3 | 8 |

**Table S3.** Library and primer sequences used in ProteoFish-SELEX platform.

| Name | Len | Sequences |
| --- | --- | --- |
| Library | 66 | CAGCACCGTCAACTGAATNNNNNNNNNNNNNNNNNNNNNNNNNNNNNNGTGATGCGATGGAGATGT |
| Forward primer | 18 | CAGCACCGTCAACTGAAT |
| Reverse primer | 18 | ACATCTCCATCGCATCAC |
| Forward primer | 18 | CAGCACCGTCAACTGAAT (5`FAM) |
| Reverse primer | 18 | ACATCTCCATCGCATCAC (5`Biotin) |

**Table S4.** Reagent concentrations used in each round of ProteoFish-SELEX.

| Round | NPs (μL) | ssDNA library into each round (pmol) | Positive (volume of serum (μL)) | Negative (volume of serum (μL)) |
| --- | --- | --- | --- | --- |
| 1 | 50 | 1000 | 50 | 50 |
| 2 | 50 | 200 | 50 | 50 |
| 3 | 50 | 150 | 50 | 50 |
| 4 | 50 | 100 | 50 | 50 |
| 5 | 50 | 75 | 50 | 50 |
| 6 | 50 | 50 | 50 | 50 |
| 7 | 50 | 50 | 50 | 50 |

**Table S5.** Sequences of the 27 differentially expressed aptamers from the last positive-SELEX of ovarian cancer.

| Name | Len | Sequences |
| --- | --- | --- |
| Seq 2 | 30 | CGCATGAGGGTCTGGCGCTTGGGTATTGGC |
| Seq 3 | 30 | GTGGGTGGGTGGGTGGTAAGTTGTGCCGAC |
| Seq 4 | 30 | GCGGGTGGGTGGGTGGTAAGTTGTGCCGAC |
| Seq 5 | 30 | GCCGTGGGTGGGAGGGTGGAACCATTCTGG |
| Seq 6 | 30 | GGATCCCCGCATCGTGGGTCAGTATTCTTC |
| Seq 7 | 30 | GGAGCCGTGGGTGGGTGGGTGGTTGCTGCC |
| Seq 8 | 29 | GGATCCTCGGCTCGTAGGTCAGTATTCTC |
| Seq 9 | 30 | GCGGTCCGTCCGAACCAGGTCTCCTGATTG |
| Seq 10 | 30 | GCGGAGGGCGGGTGGGTGGCACATTCGATG |
| Seq 11 | 30 | CACCGAGTGTGGGTGGGGGGGTGGTATTTG |
| Seq 12 | 30 | GGATCGTCGTATCGTAGGTCAGTATTCATG |
| Seq 13 | 30 | GGTATTGGGTTCGTAGAAAGGCTCCCTCTG |
| Seq 14 | 30 | CCGGGGGGCGGGTGGGCGGAGGATATCAGG |
| Seq 15 | 30 | CAGGCCGTGGGAGGGTGGGTGGTAGCCTGG |
| Seq 16 | 30 | CGCGGTGGGAGGGTGGGTGGAGCGTGGTCG |
| Seq 17 | 30 | GAGGTGGGAGGGAGGGGTTGACCTTGCGTG |
| Seq 18 | 30 | CGGCGGGTGGGTGGGTGGAGATTTAACGTC |
| Seq 20 | 30 | CAGGACCGGCGGGCGGGCGGGGGGAGGCTC |
| Seq 22 | 30 | GCGGGTGGGTGGGTGGCTCATAGTTTTTTG |
| Seq 23 | 30 | CTCGCCGCCGTTCCAGGGTATACACGTGTC |
| Seq 26 | 30 | CGTGGGTGGGTGGGTGGTTCGTGATGCGTG |
| Seq 28 | 30 | CGGCGGGTGGGTGGGCGGAGATTTAACGTC |
| Seq 29 | 30 | GAACGCACTCGCAGGGCTTGGTGTCTATGG |
| Seq 30 | 30 | GAGTCGCGGGTGGGTGGGTGGCCTTGCTCG |
| Seq 32 | 30 | CGGCGGGGGGGTGGGTGGAGATTTAACGTC |
| Seq 34 | 30 | CGGCGGGTGGGGGGGTGGAGATTTAACGTC |
| Seq 37 | 30 | TGGGAGGGGGGGTTGGGTAGTTGCGCGGAC |

**Table S6.** Sequences of the 40 differentially expressed aptamers from the last positive-SELEX of colorectal cancer.

| Name | Len | Sequences |
| --- | --- | --- |
| Seq1 | 30 | CAGAAATTCGGCCGAATAGTCACGTACCGG |
| Seq2 | 30 | CGCATGAGGGTCTGGCGCTTGGGTATCGGC |
| Seq3 | 30 | GCTAGGCCTCTACGCGGGTTCCGTCCCTTG |
| Seq4 | 30 | CGCGGGTATCCTCTGGGAGTCTGTCTTTGC |
| Seq5 | 30 | CGCATGAGGGTCTGGCGCTCGGGTATTGGC |
| Seq6 | 30 | GAACGCACTCGCAGGGCTTGGTGTCTATGG |
| Seq7 | 30 | ACGGTAACGGCGTGTGAGTGTCCTACGATC |
| Seq9 | 30 | GTGGGTGGGTGGGTGGTAAGTTGTGCCGAC |
| Seq10 | 30 | GCGGGTGGGTGGGTGGTAAGTTGTGCCGAC |
| Seq11 | 30 | GTGGGTGGGCGGGTGGATGTTGACCTCCTG |
| Seq12 | 30 | GGCTATCTCGCGCCTCCATGTGCTGTGTTC |
| Seq13 | 30 | GCGGTCCGTCCGAACCAGGTTTCCTGATTG |
| Seq14 | 30 | GCTGTGGGCCCTTCAGGTATCCTTGGGTTC |
| Seq15 | 30 | TCCGCGCCCTCCATGTGCTGCCTTTGTTTC |
| Seq16 | 30 | GGCCCACTTTCGCGCCTCCATGTGCTGTTC |
| Seq17 | 30 | CGGCGGGTGGGAGGGTGGAGATTATACGTG |
| Seq18 | 30 | ACCGCGCCTCCATGTGCTGTTCCTTTTTGC |
| Seq19 | 30 | GCGGTCCGTCCGAACCAGGTCTCCTGATTG |
| Seq20 | 30 | GGATCCCCGTTTCGCTGGTCAGTATTCTGC |
| Seq21 | 30 | GCATCGGTTATGCTCCAGGGTTCCCCGTCC |
| Seq22 | 30 | GTGCCCCGTCCGAACCAGGTTCCCTGAAGG |
| Seq23 | 30 | CCTGCGGGCGGGTGGGTGGTTGGATTCTTG |
| Seq24 | 30 | CGGCGGGTGGGTGGGCGGAGATTTAACGTC |
| Seq25 | 30 | CCTAGGGAAAGAAGTGGGCCGTTATAATGG |
| Seq26 | 29 | CGGCTAGGGACGAAGGGTCCGTTATAATG |
| Seq27 | 30 | GCCGTGGGTGGGAGGGTGGAACCATTCTGG |
| Seq28 | 30 | CGGCGGGTGGGTGGGTGGAGATTTAACGTC |
| Seq29 | 30 | GGATCCCCGCCTCGTGGGTCAGTATTCTTC |
| Seq30 | 30 | GCTGAGGGCGGGTGGGTGGCACATTCGATG |
| Seq31 | 30 | GGATCCTCGTTTCAGATGGTCAGTACTTCC |
| Seq32 | 30 | GGAGCCGTGGGTGGGTGGGTGGTTGCTGCC |
| Seq33 | 30 | GGATCCCCGCATCGTGGGTCAGTATTCTTC |
| Seq34 | 30 | GGACCTCCGTCTCGCGAGTCAGTATTCAAC |
| Seq35 | 30 | GGATCGTCGTATCGTAGGTCAGTATTCATG |
| Seq36 | 30 | CCGGGGGGCGGGTGGGCGGAGGATATCAGG |
| Seq37 | 30 | CAGGCCGTGGGAGGGTGGGTGGTAGCCTGG |
| Seq38 | 30 | GTGGCACCTGCGGTAAAGGCGACTTTTGTG |
| Seq39 | 30 | CACCGAGTGTGGGTGGGGGGGTGGTATTTG |
| Seq40 | 30 | TGGAGTGGCTTGGGTTCCCTCATTTCGTAC |
| Seq41 | 30 | CGGTAACGGCGTTTGAGTGTCATACTCTTC |

**Table S7.** Sequences of the 30 differentially expressed aptamers from the last positive-SELEX of lung cancer.

| Name | Len | Sequences |
| --- | --- | --- |
| Seq1 | 30 | CGCATAAGGGTCTGGCGCTTGGGTATTGGC |
| Seq2 | 30 | AGGGCCTGGCGATTGGGTTTGGCATGTTTC |
| Seq3 | 30 | CAGAAATTCGTCCGAATAGTCACGTACCGG |
| Seq4 | 30 | CTCGCCGCCGTTCCAGGGTATACACGTGTC |
| Seq5 | 30 | CGCATGGGGGTCTGGCGCTTGGGTATTGGC |
| Seq6 | 30 | TGCGGTAACGGCGTGTGAGTGTCTTCCCAC |
| Seq7 | 30 | TTATCGGCCCTGATAAATAGGCACCCGTTC |
| Seq8 | 30 | CAGAAATTCGGCCGAATAGTCACGTACCGG |
| Seq9 | 30 | TTGTCAACGTAGTGGGAGGGGAATTCGAGT |
| Seq10 | 30 | TGGAGTGGCTTGGGTTCCCTCATTTCGTAC |
| Seq12 | 30 | CCCTCGCGCCCTCCATGTGCTGTTTCTTTC |
| Seq13 | 30 | TGGAGTGGCTTGCGTTCCCTCATTTCGTAC |
| Seq14 | 30 | GCTGTGGGCCCTTCAGGTATCCTTGGGTTC |
| Seq15 | 30 | GAACGTACTCGCAGGGCTTGGTGTCTATGG |
| Seq16 | 30 | CCTGCGCGCCTCCATGTGCTGTTTTCTTTC |
| Seq17 | 30 | GCGGTCCGTCCGAACCAGGTTTCCTGATTG |
| Seq18 | 30 | GAACGCACTCGCAGGGCTTGGTGTCTATGG |
| Seq19 | 30 | GCGCGCCTCCATGTGCTGTATCTTTCCGTG |
| Seq20 | 30 | GCGGTCCGTCCGAACCAGGTCTCCTGATTG |
| Seq21 | 30 | GGATCCCCGTTTCGCTGGTCAGTATTCTGC |
| Seq22 | 30 | CCTAGGGAAAGAAGTGGGCCGTTATAATGG |
| Seq23 | 30 | GCATCGGTTATGCTCCAGGGTTCCCCGTCC |
| Seq24 | 30 | CGGCTAGGGACGAAGGGTCCGTTATAATG |
| Seq25 | 30 | GGACCCTCGTTTCAGAGGTCAGTATTCACC |
| Seq26 | 30 | GGATCCCCGTATCGTGGGTCAGTATTCTTC |
| Seq27 | 29 | GGATCCTCGGCTCGTAGGTCAGTATTCTC |
| Seq28 | 30 | GGATCCCCGCCTCGTGGGTCAGTATTCTTC |
| Seq29 | 30 | GGATCCCCGCATCGTGGGTCAGTATTCTTC |
| Seq30 | 29 | GGATCGTCGTATCGTAGGTCAGTATTCATG |
| Seq31 | 30 | GGATCCTCGACTCAGAGGTCAGTATTCTGC |

**Table S8.** Forty most abundant proteins in serum and NPC.

| Rank | Top 40 in serum | Percentage (%) | Top 40 in NPC | Percentage (%) |
| --- | --- | --- | --- | --- |
| 1 | Serum albumin | 40.61 | Vitronectin | 12.02 |
| 2 | Immunoglobulin gamma-1 heavy chain | 6.57 | Histidine-rich glycoprotein | 9.25 |
| 3 | Immunoglobulin kappa light chain | 3.97 | Histidine-rich glycoprotein | 6.39 |
| 4 | Apolipoprotein A-I | 3.96 | Coagulation factor XII | 5.19 |
| 5 | Serotransferrin | 3.82 | Prothrombin | 4.25 |
| 6 | Immunoglobulin heavy constant gamma 2 | 3.67 | Complement C1q subcomponent subunit B | 3.61 |
| 7 | Alpha-1-acid glycoprotein 2 | 3.25 | Kininogen-1 | 3.11 |
| 8 | Immunoglobulin kappa constant | 2.60 | Complement C1q subcomponent subunit C | 2.89 |
| 9 | Alpha-1-antitrypsin | 2.47 | Complement C1q subcomponent subunit A | 2.85 |
| 10 | Immunoglobulin lambda-1 light chain | 1.94 | Complement C3 | 2.55 |
| 11 | Hemopexin | 1.75 | Complement C4-B | 2.53 |
| 12 | Haptoglobin | 1.66 | Immunoglobulin heavy constant gamma 3 | 2.51 |
| 13 | Immunoglobulin heavy constant mu | 1.55 | Neutrophil defensin 1 | 2.46 |
| 14 | Alpha-2-macroglobulin | 1.50 | Complement C1s subcomponent | 2.21 |
| 15 | Immunoglobulin mu heavy chain | 1.27 | Apolipoprotein A-I | 1.78 |
| 16 | Immunoglobulin kappa variable 3D-7 | 1.12 | Thrombospondin-1 | 1.74 |
| 17 | Immunoglobulin heavy constant alpha 1 | 0.91 | Platelet factor 4 | 1.47 |
| 18 | Immunoglobulin alpha-2 heavy chain | 0.84 | Complement C1r subcomponent | 1.45 |
| 19 | Immunoglobulin heavy variable 3-20 | 0.62 | Tetranectin | 1.43 |
| 20 | Alpha-1-antichymotrypsin | 0.56 | Platelet factor 4 variant | 0.99 |
| 21 | Immunoglobulin heavy constant gamma 4 | 0.55 | Apolipoprotein B-100 | 0.96 |
| 22 | Apolipoprotein A-II | 0.54 | Gelsolin | 0.93 |
| 23 | Complement C3 | 0.47 | Complement factor H-related protein 1 | 0.93 |
| 24 | Alpha-1B-glycoprotein | 0.43 | Plasma kallikrein | 0.91 |
| 25 | Hemoglobin subunit beta | 0.42 | Immunoglobulin kappa constant | 0.91 |
| 26 | Immunoglobulin kappa variable 4-1 | 0.39 | Pigment epithelium-derived factor | 0.90 |
| 27 | Immunoglobulin kappa variable 3-20 | 0.38 | Insulin-like growth factor-binding protein 3 | 0.80 |
| 28 | Complement C4-B | 0.34 | Beta-2-glycoprotein 1 | 0.78 |
| 29 | Alpha-2-HS-glycoprotein | 0.32 | Keratin, type II cytoskeletal 1b | 0.75 |
| 30 | Apolipoprotein C-III | 0.31 | Complement factor H | 0.73 |
| 31 | Immunoglobulin kappa variable 3D-11 | 0.30 | Platelet basic protein | 0.72 |
| 32 | Beta-2-glycoprotein 1 | 0.28 | Apolipoprotein A-II | 0.72 |
| 33 | CD5 antigen-like | 0.27 | Immunoglobulin heavy constant alpha 2 | 0.70 |
| 34 | Immunoglobulin kappa variable 3D-20 | 0.28 | Plasma protease C1 inhibitor | 0.66 |
| 35 | Alpha-1-acid glycoprotein 1 | 0.27 | Selenoprotein P | 0.61 |
| 36 | Kininogen-1 | 0.25 | Immunoglobulin heavy constant alpha 1 | 0.58 |
| 37 | Plasminogen | 0.24 | Serum albumin | 0.56 |
| 38 | Pleckstrin homology-like domain family B member 2 | 0.24 | Immunoglobulin heavy constant mu | 0.52 |
| 39 | Ceruloplasmin | 0.22 | Charged multivesicular body protein 4a | 0.52 |
| 40 | Protein AMBP | 0.21 | Immunoglobulin heavy constant alpha 1 | 0.46 |

**Table S9.** Sequences of refined 69 unique differentially expressed aptamers across LC, OV, and CRC after deduplication.

| Name | Len | Sequences |
| --- | --- | --- |
| Seq1 | 30 | CGGCGGGTGGGTGGGTGGAGATTTAACGTC |
| Seq2 | 30 | GTGGGTGGGTGGGTGGTAAGTTGTGCCGAC |
| Seq3 | 30 | GCGGGTGGGTGGGTGGTAAGTTGTGCCGAC |
| Seq4 | 30 | GCCGTGGGTGGGAGGGTGGAACCATTCTGG |
| Seq5 | 30 | GGATCCCCGCATCGTGGGTCAGTATTCTTC |
| Seq6 | 30 | GGAGCCGTGGGTGGGTGGGTGGTTGCTGCC |
| Seq7 | 29 | GGATCCTCGGCTCGTAGGTCAGTATTCTC |
| Seq8 | 30 | GCGGTCCGTCCGAACCAGGTCTCCTGATTG |
| Seq9 | 30 | GCGGAGGGCGGGTGGGTGGCACATTCGATG |
| Seq10 | 30 | CACCGAGTGTGGGTGGGGGGGTGGTATTTG |
| Seq11 | 30 | GGATCGTCGTATCGTAGGTCAGTATTCATG |
| Seq12 | 30 | GGTATTGGGTTCGTAGAAAGGCTCCCTCTG |
| Seq13 | 30 | CCGGGGGGCGGGTGGGCGGAGGATATCAGG |
| Seq14 | 30 | CAGGCCGTGGGAGGGTGGGTGGTAGCCTGG |
| Seq15 | 30 | CGCGGTGGGAGGGTGGGTGGAGCGTGGTCG |
| Seq16 | 30 | GAGGTGGGAGGGAGGGGTTGACCTTGCGTG |
| Seq17 | 30 | CGCATGAGGGTCTGGCGCTTGGGTATTGGC |
| Seq18 | 30 | CAGGACCGGCGGGCGGGCGGGGGGAGGCTC |
| Seq19 | 30 | GCGGGTGGGTGGGTGGCTCATAGTTTTTTG |
| Seq20 | 30 | CTCGCCGCCGTTCCAGGGTATACACGTGTC |
| Seq21 | 30 | CGTGGGTGGGTGGGTGGTTCGTGATGCGTG |
| Seq22 | 30 | CGGCGGGTGGGTGGGCGGAGATTTAACGTC |
| Seq23 | 30 | GAGTCGCGGGTGGGTGGGTGGCCTTGCTCG |
| Seq24 | 30 | CGGCGGGGGGGTGGGTGGAGATTTAACGTC |
| Seq25 | 30 | TGGGAGGGGGGGTTGGGTAGTTGCGCGGAC |
| Seq26 | 30 | GAACGCACTCGCAGGGCTTGGTGTCTATGG |
| Seq27 | 30 | CGGCGGGTGGGGGGGTGGAGATTTAACGTC |
| Seq28 | 30 | CCTAGGGAAAGAAGTGGGCCGTTATAATGG |
| Seq29 | 30 | TGGAGTGGCTTGCGTTCCCTCATTTCGTAC |
| Seq30 | 30 | CAGAAATTCGGCCGAATAGTCACGTACCGG |
| Seq31 | 29 | CGGCTAGGGACGAAGGGTCCGTTATAATG |
| Seq32 | 30 | CGCAGCCGTCACTAGTCGTAGTCCCTCTGG |
| Seq33 | 30 | CGCATAAGGGTCTGGCGCTTGGGTATTGGC |
| Seq34 | 30 | CGCATGGGGGTCTGGCGCTTGGGTATTGGC |
| Seq35 | 30 | TGCGGTAACGGCGTGTGAGTGTCTTCCCAC |
| Seq36 | 30 | CCCTCGCGCCCTCCATGTGCTGTTTCTTTC |
| Seq37 | 30 | GCTGTGGGCCCTTCAGGTATCCTTGGGTTC |
| Seq38 | 30 | GCGGTCCGTCCGAACCAGGTTTCCTGATTG |
| Seq39 | 30 | GAACGTACTCGCAGGGCTTGGTGTCTATGG |
| Seq40 | 30 | GGACCCTCGTTTCAGAGGTCAGTATTCACC |
| Seq41 | 30 | TTGTCAACGTAGTGGGAGGGGAATTCGAGT |
| Seq42 | 30 | AGGGCCTGGCGATTGGGTTTGGCATGTTTC |
| Seq43 | 30 | GCATCGGTTATGCTCCAGGGTTCCCCGTCC |
| Seq44 | 30 | GGATCCCCGCCTCGTGGGTCAGTATTCTTC |
| Seq45 | 30 | GGATCCCCGTTTCGCTGGTCAGTATTCTGC |
| Seq46 | 30 | GGATCCTCGACTCAGAGGTCAGTATTCTGC |
| Seq47 | 30 | GGATCCCCGTATCGTGGGTCAGTATTCTTC |
| Seq48 | 30 | GCGCGCCTCCATGTGCTGTATCTTTCCGTG |
| Seq49 | 30 | CCTGCGCGCCTCCATGTGCTGTTTTCTTTC |
| Seq50 | 30 | CAGAAATTCGTCCGAATAGTCACGTACCGG |
| Seq51 | 30 | TTATCGGCCCTGATAAATAGGCACCCGTTC |
| Seq52 | 30 | ACGGTAACGGCGTGTGAGTGTCCTACGATC |
| Seq53 | 30 | GCTAGGCCTCTACGCGGGTTCCGTCCCTTG |
| Seq54 | 30 | CGCATGAGGGTCTGGCGCTTGGGTATCGGC |
| Seq55 | 30 | GGACCTCCGTCTCGCGAGTCAGTATTCAAC |
| Seq56 | 30 | CGCATGAGGGTCTGGCGCTCGGGTATTGGC |
| Seq57 | 30 | GTGCCCCGTCCGAACCAGGTTCCCTGAAGG |
| Seq58 | 30 | CCTGCGGGCGGGTGGGTGGTTGGATTCTTG |
| Seq59 | 30 | GTGGGTGGGCGGGTGGATGTTGACCTCCTG |
| Seq60 | 30 | GTGGCACCTGCGGTAAAGGCGACTTTTGTG |
| Seq61 | 30 | CGCGGGTATCCTCTGGGAGTCTGTCTTTGC |
| Seq62 | 30 | GGATCCTCGTTTCAGATGGTCAGTACTTCC |
| Seq63 | 30 | GCTGAGGGCGGGTGGGTGGCACATTCGATG |
| Seq64 | 30 | ACCGCGCCTCCATGTGCTGTTCCTTTTTGC |
| Seq65 | 30 | CGGCGGGTGGGAGGGTGGAGATTATACGTG |
| Seq66 | 30 | GGCTATCTCGCGCCTCCATGTGCTGTGTTC |
| Seq67 | 30 | TCCGCGCCCTCCATGTGCTGCCTTTGTTTC |
| Seq68 | 30 | GGCCCACTTTCGCGCCTCCATGTGCTGTTC |
| Seq69 | 30 | CGGTAACGGCGTTTGAGTGTCATACTCTTC |

**Table S10.** Sequences used for identification and binding affinity characterization.

| Name | Len | Sequences |
| --- | --- | --- |
| OV2 | 66 | CAGCACCGTCAACTGAATGTGGGTGGGTGGGTGGTAAGTTGTGCCGACGTGATGCGATGGAGATGT |
| OV7 | 66 | CAGCACCGTCAACTGAATTGGGAGGGGGGGTTGGGTAGTTGCGCGGACGTGATGCGATGGAGATGT |
| OV8 | 66 | CAGCACCGTCAACTGAATGAGTCGCGGGTGGGTGGGTGGCCTTGCTCGGTGATGCGATGGAGATGT |
| OV10 | 66 | CAGCACCGTCAACTGAATGAGGTGGGAGGGAGGGGTTGACCTTGCGTGGTGATGCGATGGAGATGT |
| CRC4 | 66 | CAGCACCGTCAACTGAATGCTGAGGGCGGGTGGGTGGCACATTCGATGGTGATGCGATGGAGATGT |
| CRC5 | 66 | CAGCACCGTCAACTGAATCCTGCGGGCGGGTGGGTGGTTGGATTCTTGGTGATGCGATGGAGATGT |
| CRC7 | 66 | CAGCACCGTCAACTGAATCGGCGGGTGGGTGGGCGGAGATTTAACGTCGTGATGCGATGGAGATGT |
| CRC8 | 66 | CAGCACCGTCAACTGAATGGAGCCGTGGGTGGGTGGGTGGTTGCTGCCGTGATGCGATGGAGATGT |
| CRC9 | 66 | CAGCACCGTCAACTGAATGTGGGTGGGCGGGTGGATGTTGACCTCCTGGTGATGCGATGGAGATGT |
| LC1 | 66 | CAGCACCGTCAACTGAATCAGAAATTCGGCCGAATAGTCACGTACCGGGTGATGCGATGGAGATGT |
| LC3 | 66 | CAGCACCGTCAACTGAATCGGCTAGGGACGAAGGGTCCGTTATAATGGTGATGCGATGGAGATGT |
| LC4 | 66 | CAGCACCGTCAACTGAATTGGAGTGGCTTGCGTTCCCTCATTTCGTACGTGATGCGATGGAGATGT |

1. **Supplementary references**

[1] a) W. Shi, D. Lu, L. Wang, F. Teng, J. Zhang, Core–shell structured Fe3O4@SiO2@CdS nanoparticles with enhanced visible-light photocatalytic activities. RSC Adv. 2015, 5, 106038-106043; b) J. H. Cha, H.-H. Choi, Y.-G. Jung, S.-C. Choi, G. S. An, Novel synthesis of core-shell structured Fe3O4@SiO2 nanoparticles via sodium silicate. Ceram. Int. 2020, 46, 14384-14390.

[2] M. Wang, X. Dai, X. Yang, B. Jin, Y. Xie, C. Xu, Q. Liu, L. Wang, L. Ying, W. Lu, Q. Chen, T. Fu, D. Su, Y. Liu, W. Tan, Serum Protein Fishing for Machine Learning-Boosted Diagnostic Classification of Small Nodules of Lung. *ACS Nano* **2024**, *18*, 4038-4055.
